# Neural correlates of multisensory reliability and perceptual weights emerge at early latencies during audio‐visual integration

**DOI:** 10.1111/ejn.13724

**Published:** 2017-10-25

**Authors:** Stephanie C. Boyle, Stephanie J. Kayser, Christoph Kayser

**Affiliations:** ^1^ Institute of Neuroscience and Psychology University of Glasgow Hillhead Street 58 Glasgow G12 8QB UK

**Keywords:** cross‐modal, electroencephalography, psychophysics, single‐trial analysis

## Abstract

To make accurate perceptual estimates, observers must take the reliability of sensory information into account. Despite many behavioural studies showing that subjects weight individual sensory cues in proportion to their reliabilities, it is still unclear when during a trial neuronal responses are modulated by the reliability of sensory information or when they reflect the perceptual weights attributed to each sensory input. We investigated these questions using a combination of psychophysics, EEG‐based neuroimaging and single‐trial decoding. Our results show that the weighted integration of sensory information in the brain is a dynamic process; effects of sensory reliability on task‐relevant EEG components were evident 84 ms after stimulus onset, while neural correlates of perceptual weights emerged 120 ms after stimulus onset. These neural processes had different underlying sources, arising from sensory and parietal regions, respectively. Together these results reveal the temporal dynamics of perceptual and neural audio‐visual integration and support the notion of temporally early and functionally specific multisensory processes in the brain.

## Introduction

The reliability of the information received by our senses varies. For example, visual cues become unreliable in dim or fogged conditions, and auditory cues become unreliable in loud or noisy situations. Behavioural studies have shown that observers deal with such variations in reliability by combining cues, where each is weighted in proportion to its apparent reliability (Jacobs, 1999; Ernst & Banks, 2002; Battaglia *et al*., 2003; Hillis *et al*., 2004; Helbig & Ernst, 2007; Fetsch *et al*., 2009; Butler *et al*., 2010; Raposo *et al*., 2012; Sheppard *et al*., 2013). By doing so, more reliable cues are assigned a higher weight and have stronger influence on the perceptual estimate. In most cases, this leads to a more precise and reliable percept (Ernst & Bülthoff, 2004; Ernst, 2006; Angelaki *et al*., 2009; Fetsch *et al*., 2013; Rohde *et al*., 2015).

Despite many psychophysical studies investigating the weighted combination of sensory information, the neural mechanisms underlying this process remain unclear. Single‐cell recordings have shown that neuronal sensory weights extracted from selected brain regions can vary with cue reliability in a manner consistent with predictions from statistical optimality (Gu *et al*., 2008; Morgan *et al*., 2008), and can predict perceptual weights derived from behaviour (Fetsch *et al*., 2012). Similarly, fMRI studies have demonstrated that BOLD responses are modulated by sensory reliability during visual‐tactile (Beauchamp *et al*., 2010; Helbig *et al*., 2012) and audio‐visual tasks (Rohe & Noppeney, 2016), and have shown that the sensory weighting process emerges gradually along the cortical hierarchy (Rohe & Noppeney, 2015, 2016).

While providing valuable computational insights, these studies have not determined the temporal evolution of the neural processes implementing the weighting of sensory information. Studies comparing neural response amplitudes underlying sensory integration have shown that multisensory interactions can occur at surprisingly short latencies, starting 40–76 ms after stimulus onset (Fort *et al*., 2002; Molholm *et al*., 2002; Murray *et al*., 2005, 2016; Cappe *et al*., 2010, 2012; De Meo *et al*., 2015). However, these results were obtained by comparing generic response amplitudes between uni‐ and multisensory conditions and hence did not specifically associate neural activity with either sensory reliability or a specific computational process during cue integration. Therefore, it remains unclear when following stimulus onset neuronal responses are modulated by the reliability of sensory information and when they reflect the sensory weights that drive the subsequent perceptual decision (Bizley *et al*., 2016).

In this study, we investigated these questions by examining the temporal dynamics of weighted cue combination during audio‐visual integration. We combined a rate discrimination task with EEG‐based neuroimaging, single‐trial decoding and linear modelling to identify the neural correlates of audio‐visual cue weighting. Our results show that neural activity is modulated by sensory reliability early in the trial, starting 84 ms after stimulus onset. Furthermore, we find that neural correlates of perceptual weights emerge shortly after (at 120 ms), and well before a decision is made. Finally, these EEG correlates of sensory reliability and perceptual weights localise to early sensory cortical and parietal brain areas respectively. Taken together, these results suggest that reliability‐based cue weighting computations occur early during the audio‐visual integration process, rather than later and in exclusively amodal association regions.

## Materials and methods

### Subjects

We obtained data from 20 right‐handed subjects (13 females; mean age 26 years) after written informed consent. The sample size was set to 20, based on sample sizes used in related previous EEG studies and general recommendations (Simmons *et al*., 2011). All subjects reported normal or corrected to normal vision, normal hearing and received £6 per hour for their participation. The study was approved by the local ethics committee (College of Science and Engineering, University of Glasgow) and conducted in accordance with the Declaration of Helsinki.

### Stimuli and task

**Figure 1:**
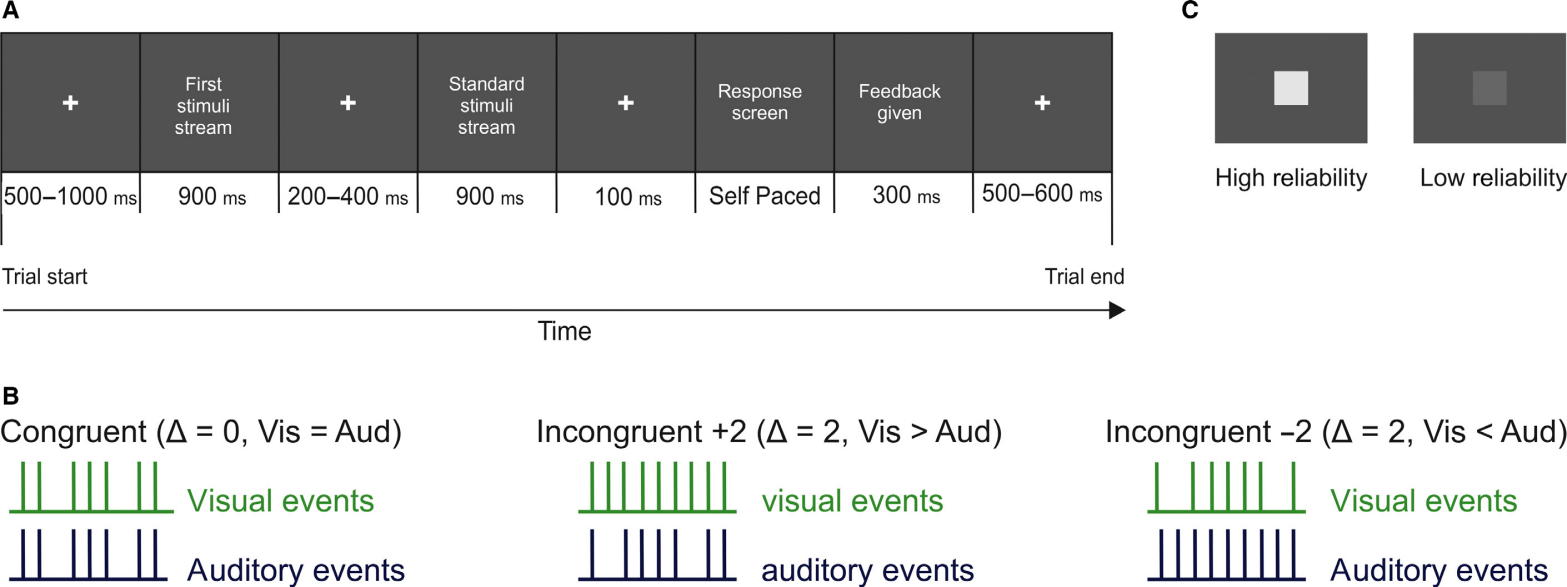
Experimental set‐up. (A) Subjects were presented with two sequential streams of auditory, visual and/or audio‐visual events and had to indicate which stream contained more events. The first stream varied in modality, event rate, reliability and congruency of the events (see Materials and methods). (B) Schematic showing one combination for each level of congruency (left: equal rates, middle: auditory fewer events, right: auditory more events). Δ = Visual − Auditory rate. (C) Example of high and low‐reliability visual stimuli. [Colour figure can be viewed at wileyonlinelibrary.com].

The task was an adapted version of a 2‐alternative forced choice rate discrimination task (Raposo *et al*., 2012; Sheppard *et al*., 2013). Subjects were presented with two streams (each lasting 900 ms) of auditory, visual or audio‐visual events (with an event defined as a single visual, auditory, or audio‐visual stimulus) and asked to decide which stream had a higher event rate (Fig. 1A). Visual events were noise squares (3 × 3 cm, 2.1° of visual angle, flashed for 12 ms each) presented atop a static pink‐noise background image. Acoustic events were brief click sounds (65 dB SPL, 12 ms duration) presented in silence. These events were instantiated by sequences of short (48 ms) or long (96 ms) pauses, causing them to appear as streams of auditory and/or visual flicker.

In the first ‘experimental’ stream, events were presented at seven different rates (8–14 Hz). In the second ‘standard’ stream events always flickered at 11 Hz. The total stream duration (900 ms) was predetermined based on the refresh rate of the computer (75 Hz) and the desired event length (12 ms). From these parameters, we calculated the possible rates that could be presented alongside the short and long pauses while staying within the 900 ms time window. Then, for each trial, short and long pauses were randomly placed around the events to create stimuli streams of equal length for all rates. For the comparison stream (where the event rate was always 11 Hz), pauses were randomly interspersed around 11 events on each trial. Important to note, on some trials the experimental stream rate was equal to the comparison stream rate. Subjects were unaware of this and were asked to make a choice about which stream had a higher event rate, which in theory could result in a bias towards inequality. To control for this potential systematic influence on behaviour, we ensured these equal stream rate trials made up only a small percentage of each block (2% of trials per condition).

Both the reliability of the visual stimulus as well as the congruency between the rates of the auditory and visual stimuli were manipulated. Placing the audio‐visual cues in conflict is necessary as it allows assessment of the degree to which subjects are biased towards each cue (Angelaki *et al*., 2009; Fetsch *et al*., 2012, 2013; Sheppard *et al*., 2013). Congruency was manipulated by introducing differences in the event rate between the auditory and visual streams. Audio‐visual trials were either congruent (Δ = 0) with auditory and visual streams each having the same number of events, or incongruent, with the visual either containing two more (Δ = +2) or two fewer (Δ = −2) events than the auditory stream (Fig. 1B). The reliability of the visual stimulus was manipulated by adjusting the contrast of the visual stimulus relative to the background (Fig. 1C). Auditory reliability was constant throughout. Manipulating the reliability of only one modality is in line with past work (Fetsch *et al*., 2012; Helbig *et al*., 2012; Rohe & Noppeney, 2016) and allowed us to keep the experiment at a reasonable length (~3 h per session) while accommodating for the additional time necessary for EEG set‐up and extended inter‐trial intervals (to include a baseline period). Contrast levels for each reliability level were derived to match individual subject's psychometric thresholds in separate calibration blocks carried out prior to the main experiment (see Experimental procedure).

These manipulations resulted in three unisensory conditions [auditory (AUD), visual high (VH) and visual low (VL)], two congruent (Δ = 0) multisensory conditions [one where both the streams were highly reliable (AVH), and one where the auditory had high and the visual low reliability (AVL)] and four incongruent audio‐visual conditions (AVH Δ = +2, AVH Δ = −2, AVL Δ = +2 and AVL Δ = −2).

### Experimental procedure

The experiment was controlled through matlab (MathWorks) using the Psychophysics Toolbox Extensions (Brainard, 1997) and custom‐written scripts. Auditory stimuli were presented using Sennheiser headphones, and visual stimuli were presented on a Hansol 2100A CRT monitor at a refresh rate of 75 Hz. All recordings were carried out in a dark and electrical shielded room.

Subjects completed two simultaneous behavioural and EEG sessions (one session per day). Each session started with two unisensory calibration blocks used to calibrate performance between auditory and visual trials. The stimuli used in these calibration blocks were the same stimuli used in the experimental blocks. However, only the easiest comparison rates (8 and 14 Hz) were used. The auditory calibration block consisted of 30 trials, with the auditory stimuli presented in silence (high reliable auditory stimuli). For auditory trials, an overall performance score was calculated. The visual calibration block consisted of 150 trials (30 trials × 5 SNRs), where the reliability of the visual stimulus varied systematically from high to low reliability. For visual trials, psychometric functions were fit to the data, and two signal‐to‐noise (SNR) levels for visual reliability were selected from the resulting psychometric curve. Visual high reliability was set as the SNR at which visual performance was equal to performance on the auditory calibration block. Visual low reliability was set at the SNR at which performance was ~30% lower than auditory performance. These reliability levels were set at the start of each experimental session and held constant throughout.

Each block of the main study consisted of 510 trials with modality (auditory, visual, audio‐visual), reliability (visual high and low), event rate (8–14 Hz) and congruency (audio‐visual Δ = 0, ±2) varying pseudo‐randomly across trials (see Stimuli and task). This created trial‐by‐trial variability in the modality, rate and reliability of the stimulus, on each trial and within each block. In total, subjects completed approximately 2040 trials over 2, 3 or 4 days depending on the subjects’ availability and level of alertness during the session (see Fig. S1A,B for individual day performance and weighting).

Each trial began with a white fixation cross presented centrally on a dark grey noise image (500–1000 ms). This was followed by the first ‘experimental’ stream (900 ms), a fixation period (200–400 ms), and then the standard stream (900 ms). After these, subjects were cued to respond using the left (‘first stream has more events’) or right (‘second stream has more events’) keyboard buttons and received feedback on their performance (Fig. 1A). For audio‐visual trials where the rate of the auditory and visual stimuli differed (i.e. Δ = ±2), feedback was generated using the average rate of the auditory and visual streams. For trials where the rates in the experimental and standard stream were equal, feedback was randomly generated. Although providing feedback is not standard practice for studies investigating reliability weighting, previous work (Raposo *et al*., 2012; Sheppard *et al*., 2013) has shown no difference in performance for subjects who received feedback compared to those who did not. Therefore, we chose to provide feedback to engage subjects with the task over the long experiment.

### EEG recording and preprocessing

EEG data were recorded using a 64‐channel BioSemi system and ActiView recording software (Biosemi, Amsterdam, Netherlands). Signals were digitised at 512 Hz and band‐pass filtered online between 0.16 and 100 Hz. Signals originating from ocular muscles were recorded from four additional electrodes placed below and at the outer canthi of each eye.

Data from individual subject blocks were preprocessed separately in matlab using the FieldTrip toolbox (Oostenveld *et al*., 2011) and custom‐written scripts. Epochs around the first stimuli stream (−1 to 2 s relative to stream onset) were extracted and filtered between 0.5 and 90 Hz (Butterworth filter) and down‐sampled to 200 Hz. Potential signal artefacts were removed using independent component analysis (ICA) as implemented in the FieldTrip toolbox (Oostenveld *et al*., 2011), and components related to typical eye blink activity or noisy electrode channels were removed. Horizontal, vertical and radial EOG signals were computed using established procedures (Keren *et al*., 2010; Hipp & Siegel, 2013), and trials during which there was a high correlation between eye movements and components in the EEG data were removed. Finally, trials with amplitudes exceeding ±120 μV were removed. Successful cleaning was verified by visual inspection of single trials. For one subject (S20), three noisy channels (FT7, P9, TP8) were interpolated using the channel repair function as implemented in the FieldTrip toolbox.

### Analysis methods

#### Psychometric performance and Bayesian integration model

For each subject, modality and stimulation rate, the proportion of ‘first stream had a higher event rate’ responses were calculated and cumulative Gaussian functions fit to the data using the psignifit toolbox for matlab (Fruend *et al*., 2011; http://psignifit.sourceforge.net/). The threshold (SD, σ) and the point of subjective equality (PSE, μ) were obtained from the best fitting function (2000 simulations via bootstrapping) and used to calculate predicted and observed perceptual weights (Fetsch *et al*., 2012). Predicted and observed weights were derived for each modality and reliability separately and averaged over congruency levels.

Predicted weights reflect the weights that a Bayesian optimal observer would assign to each sensory cue in multisensory conditions (Fetsch *et al*., 2012). These were calculated using the thresholds (σ) from unisensory trials:

(1) $$W_{\mathrm{AUD}}=\frac{1/\sigma_{\mathrm{AUD}}^{2}}{\left[1/\sigma_{\mathrm{AUD}}^{2}+1/\sigma_{\mathrm{VIS}}^{2}\right]}.$$

Observed perceptual weights represent the apparent weight a subject assigns to each sensory cue. These were calculated from the PSE (μ) from multisensory trials:

(2) $$W_{\mathrm{AUD}}=\frac{\left[\mu_{\mathrm{AV}(\Delta )}-\mu_{\mathrm{AV}(\Delta =0)}+\Delta /2\right]}{\Delta },$$

where Δ represents the incongruency between the auditory and visual stimuli (Fetsch *et al*., 2012). For both perceptual and observed weights, we assumed that auditory and visual weights sum to one:

$$W_{\mathrm{VIS}}=1-W_{\mathrm{AUD}}.$$

#### Time‐dependent perceptual weights

We used logistic regression to model the relationship between sensory evidence and behavioural reports at each time point. This allowed us to examine how perceptual weights evolved over the course of a trial. As a measure of sensory evidence, we used the accumulated rate, defined as the number of stimulus events presented up to each time point in the trial. Accumulated rate was calculated in 12 ms time bins (our stimuli were each presented for 12 ms), resulting in 75 time points for the full stimulus stream of 900 ms. Accumulated rate was regressed against behavioural choice (first stream higher event rate vs. second stream higher event rate) for each trial, condition (AVH auditory, AVH visual, AVL auditory and AVL visual) and time point separately, yielding a trial and time‐specific measure of the experienced sensory evidence. This analysis was restricted to incongruent audio‐visual trials and a time window from 24 to 600 ms post‐stimulus onset to account for null values (pre‐24 ms) and multicollinearity in the predictor matrix (post‐600 ms).

To assess whether the accumulated rate was a significant predictor of perceptual choice, we quantified the predictive performance of the regression model (referred to as Az) using the area under the receiver operator characteristic (ROC) and 10‐fold cross‐validation (see Statistics). To determine how well the perceptual weights derived from the psychometric curves corresponded to the perceptual weights derived from the regression model, we also computed the correlation between the reliability influence for each pair of weights (psychometric and regression) at each time point during the trial (see Statistics). The reliability influence was here defined as the difference (D) in auditory and visual weights [*W*_AUD_ − *W*_VIS_] across reliability levels [AVH, AVL] at each time point (t) (in other words, the effect of visual reliability on auditory weights):

(3) $$D\left(t\right)=\left[{\text{AVH}}_{W_{\mathrm{AUD}}}-{\text{AVH}}_{W_{\mathrm{VIS}}}\right]-\left[{\text{AVL}}_{W_{\mathrm{AUD}}}-{\text{AVL}}_{W_{\mathrm{VIS}}}\right].$$

### Single‐trial EEG analysis

We used single‐trial, multivariate linear discriminant analysis (Parra *et al*., 2005; Philiastides & Sajda, 2006; Ratcliff *et al*., 2009; Philiastides *et al*., 2014; Kayser *et al*., 2016) to uncover EEG components that best discriminated between our two conditions of interest. We trained our classifier to discriminate between high and low stimulus rates (i.e. whether the first stream had an event rate that was lower or higher than the standard stream of 11 Hz) as this reflected the task the subjects were asked to complete. This analysis generated a one‐dimensional projection (*Y*_t_) of the multidimensional EEG data (*X*_t_), defined by spatial weights (*W*_t_) and a constant (C):

(4) $$Y_{\left(t\right)}=W_{\left(t\right)}X_{\left(t\right)}+C,$$

where the weight vector (*W*) represents the activity components most sensitive to the sensory stimuli, and the discriminant output (*Y*) provides a neural signature of the quality of the single‐trial evidence about the condition of interest. This approach preserves the trial‐to‐trial variability of the data and is assumed to be a better estimator of the underlying single‐trial task‐relevant activity than the data on individual channels (Parra *et al*., 2005; Blankertz *et al*., 2011; Philiastides *et al*., 2014; Kayser *et al*., 2016).

Classification was based on regularised linear discriminant analysis (Philiastides *et al*., 2014) and applied to the EEG activity at each 5 ms time point from stimulus onset to 600 ms post‐stimulus onset in sliding time windows of 55 ms. For each time point, the EEG data within the 55 ms window were averaged and the discriminant output (*Y*) aligned to the onset of the 55 ms window. This generated a sensory matrix (trials × time) that represented the information about stimulus rate in neural signals over time. To avoid introducing bias to either sensory modality, we derived the weighting vector (*W*_t_) and constant (C) from the congruent audio‐visual trials only (AVH and AVL Δ = 0) and applied these to all other trials at the same time point. Scalp topographies for the discriminating component were estimated via the forward model (Philiastides *et al*., 2014), defined as the normalised correlation between the discriminant output and the EEG activity.

### Neural weights

To quantify the apparent weight with which the sensory information in each modality contributed to the discriminant output (*Y*), we used linear regression. Similar to the behavioural data, the trial‐specific accumulated rates were used as predictors and regressed against the discriminant output (*Y*) at each time point in the trial where the classifier performed significantly (24–400 ms). Again, this analysis was restricted to incongruent audio‐visual conditions (AVH and AVL Δ ±2) and separate weights for each modality in the high‐ and low‐reliability conditions were generated. This resulted in four neural weights for each time point: one for AVH auditory, AVH visual, AVL auditory and AVL visual. As the precise neural origin of these EEG discriminant output components (and hence the respective generators of auditory and visual contributions to these) remains unclear, we did not assume that auditory and visual neural weights (for each reliability) normalised to a fixed sum of one. Consequently, we did not perform normalisation on the regression weights. This follows previous electrophysiological studies on the neural mechanisms of sensory integration (Fetsch *et al*., 2012). Finally, to assess the relationship between these neural weights and the time‐dependent perceptual weights, we correlated the reliability influence (Eqn (3)) between these at each time point (see Statistics).

### Source localisation

To obtain a confirmatory representation of the neural generators underlying the sensory representations extracted by the discriminant analysis, we used source localisation. This involved correlating the source signals with the discriminant output (*Y*) and is comparable to obtaining forward scalp models from linear discriminant analysis (Parra *et al*., 2005).

The standard Montreal Neurological Institute (MNI) magnetic resonance imaging (MRI) template was used as the head model and interpolated with the AAL (automated anatomical labelling) atlas. Leadfield computation was based on the standard source model (3D grid model with 6 mm spacing) and a manually aligned BioSemi electrode channel template. Covariance matrices were calculated from −200 ms pre‐stimulus onset to 800 ms post‐stimulus onset and source localisation performed on individual subject single‐trial data using a linear constrained minimum variance beamformer in Fieldtrip (fixed orientation, 7% normalisation). This resulted in 11 432 unique grid points within the brain for which the source signal was extracted. Source signals were then correlated with the discriminant output (*Y*), for each subject and time point within our analysis window (24–400 ms) separately, and correlation signals z‐transformed and averaged over subjects.

### Statistics

All descriptive statistics reported represent median values. All *Z* values reported were generated from a two‐sided Wilcoxon signed‐rank test after testing assumptions of normality, and effect sizes calculated by dividing the *Z* value by the square root of *N* (where *N* = the number of observations rather than subjects). Correlations were calculated using Spearman rank correlation analysis. All reported *P*‐values were checked for inconsistencies using the R software package ‘statcheck’ (Nuijten *et al*., 2016).

Significance levels of classification performance (Az) were determined by randomly shuffling the data by condition 2000 times, computing the group averaged Az value for each randomisation and taking the maximal Az value over time to correct for multiple comparisons (referred to as randomisation test in text). This generated a distribution of group averaged Az values based on 2000 randomised data sets, from which we could estimate the Az value leading to a significance level of *P* < 0.01. For all analyses, exact *P*‐values are reported in the corresponding tables.

Significant clusters for all other comparisons were determined using a cluster‐based randomisation technique (referred to as cluster randomisation in text, Maris & Oostenveld, 2007). Data were shuffled randomly across conditions, and for each separate comparison, a distribution of *t*‐values based on 1000 randomisations was computed. For all comparisons we used a cluster‐threshold of *t* = 1.8, minimum cluster size of 2 and the max‐size as cluster‐forming variable. Effect sizes were indicated as the equivalent *r* value that is bounded between 0 and 1 (Rosenthal & Rubin, 2003).

To improve readability in the following results section, significance thresholds are reported within text while exact *P*‐values and statistics are reported in corresponding tables.

## Results

### Psychometric behaviour and perceptual thresholds

Subjects’ performance was quantified by fitting behavioural performance (‘first stream higher’ responses) with psychometric curves to derive psychometric thresholds (σ) and points of subjective equality (PSE, μ). From these measures, a set of predicted and observed perceptual weights were generated.

**Figure 2:**
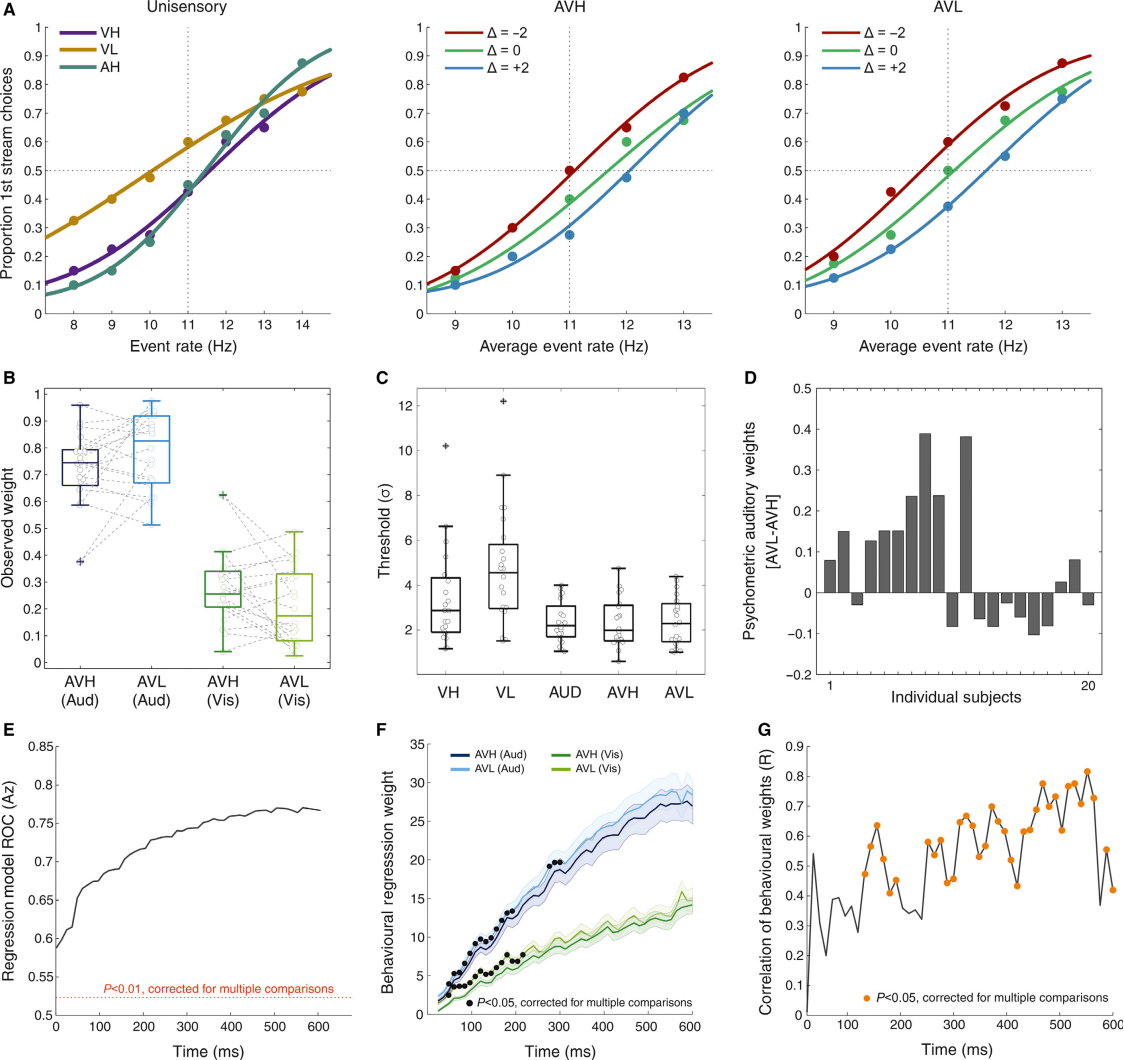
Behavioural results. (A) Group (*n* = 20) level psychometric curves are displayed as the proportion of ‘first stream’ decisions as a function of event rate for each condition. Note that for incongruent trials, the *x*‐axis indicates the average event rate (Δ = Visual Rate − Auditory rate). Vertical dashed lines represent the standard rate (11 Hz) and horizontal dashed lines represent chance (50%) performance. (B) Observed perceptual weights with individual subject data shown in grey. AVH represents the audio‐visual condition where both the auditory and visual cues were equally reliable. AVL represents the audio‐visual condition where the auditory was highly reliable and the visual was less reliable. (C) Individual subject threshold values (σ) for each separate condition. (D) The difference between auditory weights in the AVH and AVL conditions (*W*_AVL_ − *W*_AVH_) for each subject. (E–F) Logistic regression was used to predict single‐trial choice (event rate >/<11 Hz) based on the accumulated event rate at each time point in the trial. Shaded error bars represent standard error of the mean. (E) Performance of the logistic model quantified using the area under the ROC (dashed line *P* < 0.01, randomisation test) (F) Auditory and visual perceptual weights derived from the regression model. Time points with significant reliability effects are denoted with black circles. (G) Correlation of perceptual weights derived from psychometric curves and from the logistic model. Time points with significant correlations are marked with orange circles. [Colour figure can be viewed at wileyonlinelibrary.com].

Figure 2A shows the group‐level psychometric curves for each sensory condition. On unisensory trials, thresholds were significantly lower (i.e. better performance) for high compared to low‐reliability stimuli across subjects (*P* < 0.05, Table 1, Fig. 2C). Thresholds were comparable for the auditory and both congruent audio‐visual conditions (*P* > 0.05, Table 1). Thresholds on audio‐visual trials were significantly lower compared to the visual conditions (*P* < 0.05, Table 1). This demonstrates that performance was comparable on audio‐visual and auditory trials, lowest for visual trials and better for high vs. low reliable stimuli.

**Table 1:** Analysis of psychometric data

Psychometric fits			Comparison of perceptual thresholds			
	Median σ	Median μ		*Z* value	*P*‐value	Effect size
AUD	2.19	11.45	VH vs. AUD	−2.688	0.007	−0.425
VH	2.87	11.44	VL vs. AUD	−3.658	0.0003	−0.579
VL	4.55	10.53	VH vs. VL	−3.136	0.002	−0.496
AVH (Δ = 0)	1.98	11.72	AUD vs. AVH	−0.336	0.737	−0.053
AVH (Δ = +2)	1.80	12.25	AUD vs. AVL	−0.261	0.794	−0.041
AVH (Δ = −2)	1.89	11.05	AVH vs. AVL	−0.018	0.986	−0.003
AVL (Δ = 0)	2.29	11.14	AVH vs. VH	3.322	0.0009	0.525
AVL (Δ = +2)	1.82	11.94	AVH vs. VL	3.621	0.0003	0.573
AVL (Δ = −2)	2.10	10.58	AVL vs. VH	3.397	0.0007	0.535
			AVL vs. VL	3.919	0.00009	0.619

Median threshold (σ) and PSE (μ) values from fits to psychometric data (left). Statistical tests (right) were based on two‐sided Wilcoxon signed‐rank tests of thresholds (σ).

Comparing psychometric curves for congruent and incongruent multisensory conditions showed that regardless of visual reliability subjects preferentially weighted the auditory modality. This is demonstrated by shifts in the psychometric curves towards the auditory rate (Fig. 2A left and right). However – as expected – this shift was more pronounced in the low‐reliability condition, showing a stronger influence of the auditory modality when visual reliability was reduced (Table 1).

Predicted auditory weights significantly increased when visual reliability was reduced (*P* < 0.05, Table 2). However, this pattern was not consistently found in the observed weights (Fig. 2B), where there was no significant difference between observed auditory weights between reliabilities (*P* > 0.05, Table 2). Furthermore, a direct comparison between observed and predicted weights revealed only a weak correlation (AVH *P* > 0.05, AVL *P* < 0.05, Table 2).

**Table 2:** Analysis of predicted and observed psychometric weights

High‐ vs. Low‐reliability psychometric (auditory) weights				Predicted vs. Observed psychometric auditory weights		
	*Z*	*P*‐value	Effect size		*R*_s_	*P*‐value
Pred AVH vs. Pred AVL	−2.837	0.005	−0.454	Pred AVH vs. Obs AVH	0.21	0.381
Obs AVH vs. Obs AVL	−1.261	0.207	−0.199	Pred AVL vs. Obs AVL	0.48	0.034

Comparison of auditory weights based on two‐sided Wilcoxon signed‐rank tests (left; High‐ vs. Low‐reliability comparisons) and Spearman rank correlations (right; *R*_s_, Predicted vs. Observed comparisons).

The lack of a significant difference between unisensory and multisensory thresholds and the weak correlation between observed and predicted weights suggests that observers did not systematically follow the behavioural pattern predicted by Bayesian models of multisensory integration. This is corroborated by Fig. 2D, which shows the magnitude and direction of the weight shift across subjects. Only 11 subjects showed a shift of auditory weight shifts in the predicted direction (i.e. increased auditory weighting when visual reliability is reduced). For the present study, this heterogeneity in the change of perceptual weights with reliability presented a unique opportunity to investigate the neural correlates of perceptual weights independently of an effect of sensory reliability, as these two effects are dissociable across subjects.

### Evolution of perceptual weights over time

To obtain insights into the temporal dynamics of the perceptual weighting process, we modelled the relationship between behavioural choice and sensory evidence at each time point within a trial and derived a set of dynamic perceptual weights for each modality and reliability condition. Importantly, having a time‐resolved measure of perceptual weights allowed us to test when during a trial these changed with sensory reliability and to map behavioural weights onto similarly time‐resolved neural weights.

We found that the sensory evidence (accumulated rate) was significantly predictive of behavioural choice across the trial (permutation test, *P* < 0.01, Fig. 2E). Auditory and visual perceptual weights increased as sensory evidence was accumulated throughout the trial and confirmed that subjects preferentially weighted the auditory over the visual modality (cluster randomisation tests, *P* < 0.05, Fig. 2F, Table 3). We found that both auditory and visual weights changed significantly with reliability (cluster randomisation tests, *P* < 0.05, Fig. 2F), and did so early during the trial [two auditory clusters (48–192; 276–300 ms), one visual cluster (48–216 ms), Table 3]. To test how consistent these time‐resolved perceptual weights were with those derived from the psychometric curves, we computed their correlation. Significant correlations (cluster randomisation tests, *P* < 0.05, Fig. 2G) emerged during three epochs that collectively covered most of the trial (three clusters: 132–192, 252–408 and 420–600 ms, Table 3).

**Table 3:** Statistical effects for comparisons of perceptual or neural weights between conditions

	Cluster #	Time (ms)	*P*‐value	*t*‐value	Effect size
Time‐resolved perceptual weights					
AH vs. AL	1	48–192	< 0.0001	−13	0.5005
	2	276–300	0.010	−3	0.5604
VH vs. VL	1	48–216	< 0.0001	−15	0.5976
PMC vs. PRW*	1	48–588	< 0.0001	6	0.5201
Neural weights: Modality dominance					
AH vs. VH	1	36–60	< 0.001	3	0.5576
	2	108–120	< 0.001	2	0.5055
	3	252–264	< 0.001	−2	0.4566
AL vs. VL	1	60–96	< 0.001	11	0.5564
	2	120–336	< 0.001	2	0.5014
Neural weights: Sensory reliability					
AH vs. AL	1	156–204	< 0.0001	−5	0.4940
	2	264–276	0.0070	−2	0.4731
VH vs. VL	1	84–108	< 0.0001	3	0.5139
	2	252–288	< 0.0001	4	0.5406
Neural vs. Perceptual weights					
NW vs. PRW*	1	120–132	0.005	2	0.4674
	2	204–228	< 0.0001	3	0.5170

Test was performed using cluster permutations statistics (see Statistics). For each significant effect, we list cluster *P*‐value (where *P*‐values below 10^−3^ are listed as < 0.001), cluster *t*‐values and effect size. *t*‐values marked with * reflect correlations. PMC, psychometric curve weight; PRW, perceptual regression weight; NW, neural weight. Condition abbreviations (AUD, VH, VL, AVH, AVL) see: Materials and methods.

### EEG signatures of event rates

We used linear discriminant analysis to extract EEG components that maximally discriminated between event rates (</> 11 Hz). Such an approach allowed the use of the discriminant output (*Y*) as a proxy to the single‐trial stimulus evidence reflected in the EEG activity (Ratcliff *et al*., 2009; Philiastides *et al*., 2014; Kayser *et al*., 2016), and we exploited this to link the neural signature of the sensory input to changes in the external sensory reliability and perceptual weights. To assess how this neural signature of the event rate was modulated by sensory reliability, we regressed the discriminant output (*Y*) against the accumulated rate to obtain neural sensory weights. We restricted this analysis to the time window where the classifier could significantly discriminate between conditions (0–400 ms).

**Figure 3:**
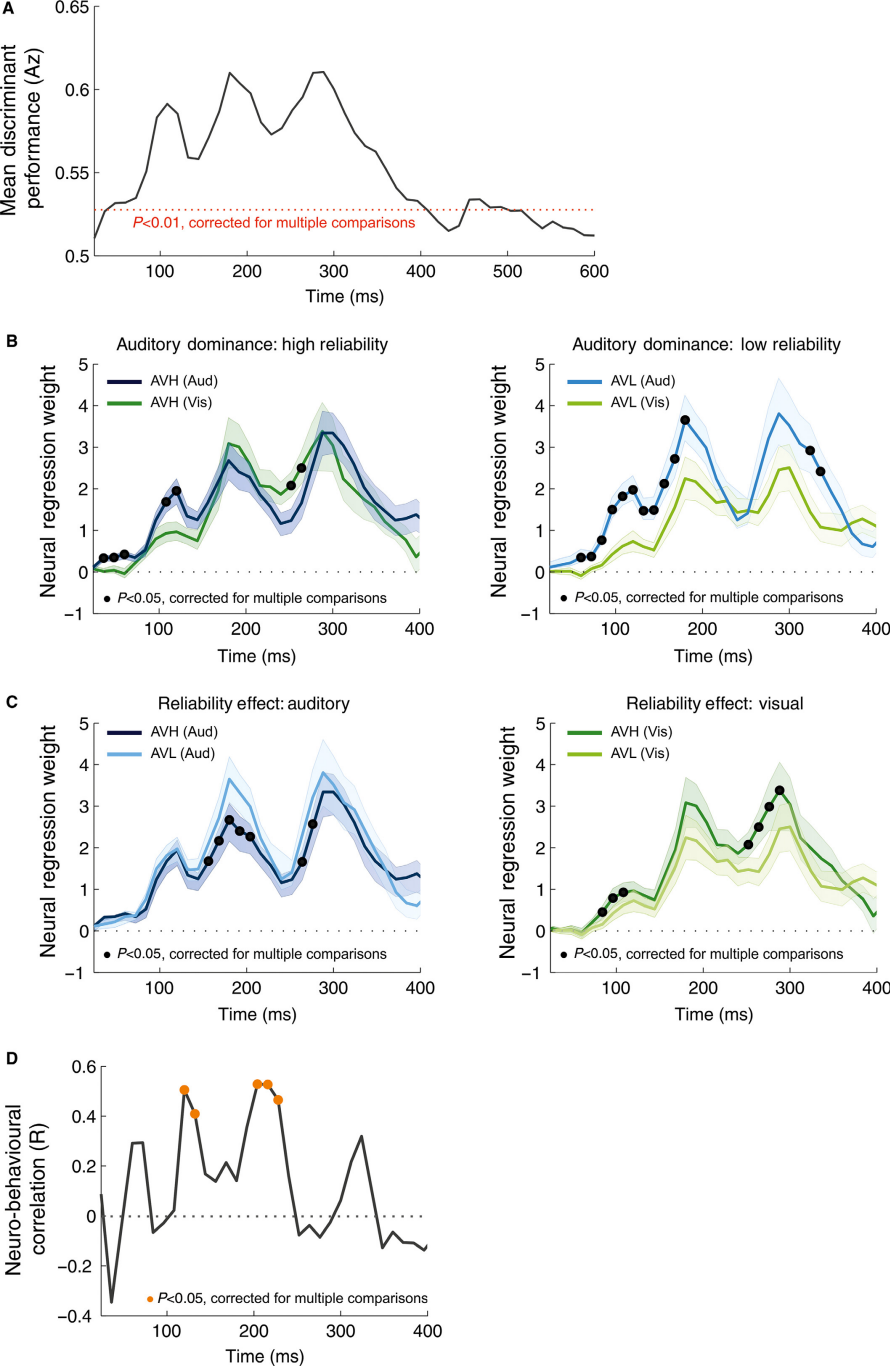
EEG decoding, neural weights and neuro‐behavioural correlation. (A) Group averaged performance of a linear classifier discriminating between the two stimulus conditions (event rate >/<11 Hz) quantified using the area under the ROC curve. The discriminant output (*Y*) was calculated using a sliding time window of 55 ms aligned to the window onset, from 24 to 600 ms post‐stimulus onset. (B) Neural weights for each modality for high reliability (left) and low‐reliability trials (right). (C) Neural weights for each reliability for auditory (left) and visual stimuli (right). In each panel (B,C), time points with significant reliability/modality effects are indicated by black circles and shaded error bars represent standard error of the mean. (D) Neuro‐behavioural correlation between the perceptual and neural weights obtained from the regression models. Time points with significant correlations are indicated by orange circles. [Colour figure can be viewed at wileyonlinelibrary.com].

Figure 3A displays the discriminant performance across subjects. Significant performance emerged early in the trial (48–396 ms, permutation test, *P* < 0.01) and was highest during three time epochs (96–120, 168–204 and 252–288 ms, with peaks defined as Az performance > 0.58).

### Influence of reliability on neural weights

First, we determined whether the neural weights exhibited a similar bias towards the auditory modality as the perceptual weights did. Indeed, auditory weights dominated in both reliability conditions (Fig. 3B left and right), and these differences emerged at several epochs across the trial (three clusters AVH: 36–60, 108–120 and 252–264 ms; two clusters AVL: 60–96 and 120–336 ms; cluster randomisation tests, *P* < 0.05, Table 3). Second, we quantified how neural weights were affected by sensory reliability. Consistent with perceptual weights, auditory weights were significantly higher when the visual reliability was reduced, during two epochs (156–204 and 264–276 ms; cluster randomisation test, *P* < 0.05, Fig. 3C left, Table 3). Visual weights were significantly lower when the visual reliability was reduced at two epochs (84–108 and 252–288 ms; cluster randomisation test, *P* < 0.05, Fig. 3C right, Table 3). Third, we asked whether there was a significant relationship between the reliability effect on the time‐resolved perceptual and the neural weights. This revealed two epochs during which reliability effects correlated significantly: 120–132 and 204–228 ms (cluster randomisation test, *P* < 0.05, Fig. 3D, Table 3).

Summarising the above results in order of time (rather than by statistical contrast) shows that there is an evolving pattern of neural weights as the trial progresses. Starting from stimulus onset, we first observe a change in visual weights (starting 84 ms) and a significant relationship between perceptual and neural weights (starting 120 ms). This is followed by a change in auditory weights (starting 156 ms) and another epoch where there is a significant relationship between perceptual and neural weights (starting 204 ms). Finally, there is a change in both visual (starting 252 ms) and auditory weights (starting 264 ms) later in the trial.

These three statistical contrasts (auditory, AVH vs AVL; visual, AVH vs AVL; and perceptual vs. neural weight difference) revealed six epochs during which neural weights exhibited patterns of interest in relation to our main questions. To disentangle whether these epochs represent distinct neural processes or whether these related to the same underlying neural generators, we analysed the relationship between these effects further. We did so by comparing scalp projections and neural weights between the six epochs (Fig. S2). This revealed that temporally adjacent topographies (84–108 and 120–132 ms; 156–204 and 204–228 ms; and 252–288 and 264–276 ms) were highly correlated (within Epochs: *R*_S_ > 0.6, *P* < 0.005 for all comparisons). The reliability difference in neural weights (Eqn (3)) at temporally adjacent epochs was also highly correlated (*R*_s_ > 0.6, *P* < 0.001) and showed similar patterns of neural weights. Consequently, we concatenated the six epochs based on their high correlations into three separate epochs of interest (Epoch 1: 84–132 ms; Epoch 2: 156–228 ms; and Epoch 3: 252–276 ms).

### Localisation of neural sources

**Figure 4:**
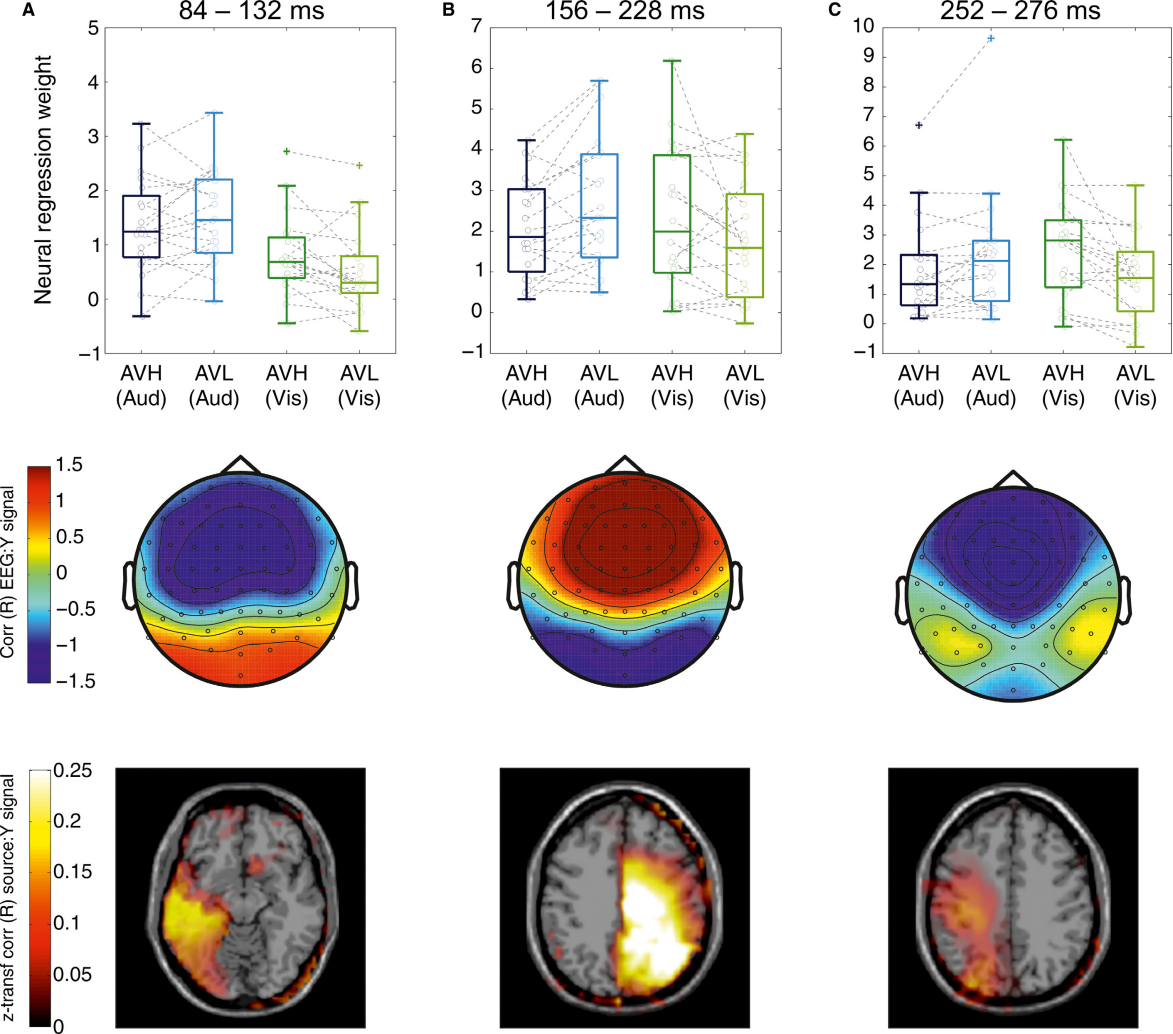
Neural Weights, Topographies and Source Localisation results for three EEG components of interest. Each component was defined based on the statistical contrast between sensory reliabilities (Fig. 3), or a significant neuro‐behavioural (N2B) correlation (Fig. 3D). In each panel, boxplots represent neural weights averaged over each epoch, with individual subject data in grey. Topographies represent the group averaged forward models averaged over the epoch, where the values represent the correlation between the discriminating output (*Y*) and the underlying EEG activity. Source maps represent the *z*‐transformed correlation values of single‐voxel activity with the discriminant output during the *t* time epochs defined by the three components of interest (left hemisphere on left‐hand side. Source localisation *z*‐coordinates for slice: (A) −7, (B) 37 and (C) 51). [Colour figure can be viewed at wileyonlinelibrary.com].

Figure 4 shows the respective neural weights, forward model scalp topographies and source localisation maps for these three epochs of interest. Table 4 reports coordinates and statistical values for significant voxels. The first epoch (84–132 ms) was characterised by a scalp projection revealing strong contributions of occipital electrodes, consistent with a potential origin in sensory cortices. Source localisation revealed that discriminant activity originated from occipital and temporal regions. The second epoch (156–228 ms) had a scalp projection that revealed strong contributions from fronto‐central electrodes, and source activity was broadly localised to temporal, occipital and parietal regions. Finally, the third epoch (252–276 ms) revealed contributions from temporal and central electrodes with sources in occipital and parietal regions.

**Table 4:** Source localisation of discriminant activity

	Co‐ord (spm)	*z*‐transformed *R*	*P*‐value	*t*‐value	Effect size
Epoch 1: 84–132 ms					
Occipital mid L/Inf. L	(−45,−70,0)	0.145	0.004	−6.949	0.486
Temporal mid. L.	(−60,−38,−10)	0.197	0.004	−7.687	0.526
Epoch 2: 156–228 ms					
Occipital Mid. R.	(37,−72,27)	0.266	< 0.001	−8.336	0.541
Temporal Inf. R.	(58,−15,−25)	0.267	< 0.001	−6.768	0.476
Parietal Inf. R.	(47,−57,41)	0.268	< 0.001	10.275	0.439
Epoch 3: 252–276 ms					
Occipital Mid. L.	(−35,−86,18)	0.166	0.003	7.575	0.416
Parietal L.	(−36,−32,46)	0.159	< 0.001	12.223	0.499

Significance values obtained from correlating the source signals with the discriminant output (*Y*) corrected for multiple comparisons using cluster permutation testing (see Statistics). For each significant source, the table provides coordinates of peak voxel (co‐ord spm), z‐transformed *R* value (as plotted in Fig. 4, bottom row), and the *P*‐value, cluster *t*‐value and effect size.

## Discussion

This study examined the temporal dynamics of reliability‐based cue weighting during an audio‐visual rate discrimination task. Our results revealed three epochs during which brain activity exhibited correlates of sensory reliability or the behaviourally attributed perceptual weights. Specifically, both the perceptual and neural weights were modulated by the reliability of sensory information as early as 84 ms after stimulus onset, and neural weights correlated with perceptual weights around 120 ms. Together, these results demonstrate that the weighted combination of sensory information arises early after stimulus onset and within sensory and parietal regions, rather than late and in amodal association cortices.

### Perceptual weights

#### Evolution over time

We performed two analyses to generate perceptual weights. The first followed the conventional approach of calculating weights based on psychometric curves fit to subjects’ responses, while the second used regression modelling to generate a set of perceptual weights based on the time‐varying accumulated sensory evidence. This dual approach allowed different views of the reliability effects on behavioural weights and allowed us to apply comparable analyses to behavioural and neural data (Gu *et al*., 2008; Fetsch *et al*., 2012).

These two approaches revealed divergent results regarding the effect of sensory reliability on perceptual weights. The analysis of psychometric curves revealed no significant group effect of reliability, while the time‐resolved behavioural weights were significantly modulated by reliability in the direction as expected by the literature: auditory weights increased as visual weights decreased (Rosas *et al*., 2005; Helbig & Ernst, 2007; Fetsch *et al*., 2009; Raposo *et al*., 2012; Sheppard *et al*., 2013). One potential explanation for this apparent discrepancy is methodological. The psychometric performance is measured only at the end of the trial and is based on trial labels, while the regression analysis quantifies choice based on the time‐aggregated sensory evidence. Hence, one possibility is that early sensory information may contribute to perceptual weighting in a more specific manner than the average sensory information available throughout the trial. Alternatively, the difference in the results obtained by the two approaches may be more gradual (with one being possibly more sensitive and producing stronger statistical significance) rather than being conceptual; this is corroborated by our finding of a significant correlation between the reliability effects for each set of perceptual weights, which suggests there is overall similarity between the two sets.

#### Auditory bias

Our group data demonstrate that subjects failed to show the expected multisensory benefit, as shown by comparable performance on audio‐visual and auditory trials. We also observed a general bias towards the auditory stimulus regardless of visual reliability in the majority of subjects. This bias emerged despite efforts to equalise visual and auditory thresholds using unisensory calibration blocks and caused a mismatch between the predicted and observed perceptual weights. A simple potential contributing factor could be that the auditory stimulus was presented in silence while the visual stimulus was embedded in noise, and this may have introduced subtle differences in intramodal attention (Alho *et al*., 1992; Lu & Dosher, 1998). In our case, however, it is likely that the observed auditory bias arises from the preference for auditory over visual information for temporal judgements (Glenberg & Fernandez, 1988; Glenberg *et al*., 1989; Repp & Penel, 2002; Recanzone, 2003) as our task was a rate discrimination task. This could potentially manifest itself by subjects performing as well on auditory trials as audio‐visual trials, thus generating a bias towards the auditory modality on audio‐visual trials. Similar modality biases have been reported in the literature before (Battaglia *et al*., 2003; Knill & Saunders, 2003; Oruç *et al*., 2003; Rosas *et al*., 2005, 2007; Fetsch *et al*., 2009, 2012; Butler *et al*., 2010; Sheppard *et al*., 2013), and it has been shown that introducing modality specific priors into Bayesian models can provide better fits to the behavioural data. For example, Battaglia *et al*. (2003) examined cue weighting using a spatial localisation task and found that both the reliability of a visual stimulus and a bias for visual information over auditory affected the perceptual weights. They showed that a ‘hybrid model’ – which included a prior to make greater use of the visual information – provided a better fit to the data than the standard integration model. Butler *et al*. (2010) made similar findings. Taken together alongside our results, these findings suggest that including a prior to account for modality bias could improve the predictions of optimal integration models. Adapting such a model was however beyond the scope of this project, and so we chose to exploit the mismatch between sensory reliability and perceptual weighting to study the neural correlates underlying both processes.

Another possibility for the observed auditory bias is that subjects did not consider auditory and visual stimuli to originate from the same underlying sensory cause, in particular on trials where event rates differed. Such stimulus‐dependent changes in the inference about the causal structure of the environment have recently been included in models of sensory integration (Shams *et al*., 2005; Roach *et al*., 2006; Knill, 2007; Körding *et al*., 2007; Beierholm *et al*., 2009; see Shams & Beierholm, 2010; Kayser & Shams, 2015 for reviews), and recently neuroimaging studies have started to investigate the neural mechanisms underlying this flexibility in sensory integration (Rohe & Noppeney, 2015, 2016). However, given that the present experiment included only one level of audio‐visual discrepancy, it is not possible to ascertain whether causal inference processes contribute to the apparent mismatch between the observed psychometric performance and predictions based on optimal integration strategies. While fitting such a model is beyond the scope of this project, it provides an interesting starting point for future work.

### Decoding stimulus rate from EEG activity

We used linear discriminant analysis to extract task‐relevant EEG components from the EEG activity and examined the influence of sensory reliability on these neural signatures. Our results thereby add to the growing literature that uses multivariate single‐trial decoding of EEG data to reveal the dynamic representation of various types of sensory stimuli (Philiastides & Sajda, 2006; Ratcliff *et al*., 2009; Wyart *et al*., 2012; Lou *et al*., 2014; Philiastides *et al*., 2014; Mostert *et al*., 2015; Kayser *et al*., 2016). In addition, we exploit this approach to quantify multisensory interactions directly within those EEG components carrying the relevant sensory representations rather than relying on generic stimulus‐related evoked responses.

Our results showed the classifier was not able to successfully decode stimulus rate later than 400 ms following stimulus onset. There are two possible explanations for this: either sensory rate is only linearly reflected in EEG activity early during the trial or neural activity later in the trial reflects a mix of sensory encoding and decision‐making processes, which may make it difficult to extract purely sensory representations (Raposo *et al*., 2014). While this precludes us from making statements about the patterns of sensory weighting that may occur later in the trial, our results directly reveal neural correlates of sensory reliability and of perceptual weights are evident early during the integration process.

### Neural correlates of sensory reliability and perceptual weights

Our results demonstrate neural correlates of both sensory reliability and perceptual weights at multiple times early during a trial. We thereby step beyond previous neurophysiological (Gu *et al*., 2008; Morgan *et al*., 2008; Fetsch *et al*., 2012) and neuroimaging studies (Beauchamp *et al*., 2010; Helbig *et al*., 2012; Rohe & Noppeney, 2015, 2016) by revealing the temporal evolution of the sensory weighting process in functionally specific brain activity. In addition, by dissociating the influence of sensory reliability from perceptual weighting in EEG responses, rather than demonstrating a simple modulation of evoked response amplitudes, we show that these early effects reflect sensory and computationally specific processes.

#### Early effects of sensory reliability

We found that early during the trial neural sensory weights scaled with reliability. At the earliest time (84–132 ms), these effects were associated with changes in visual weights, while in a slightly later window (starting at 156 ms), auditory neural weights scaled with changes in visual reliability. Even later in the trial (starting at 252 ms), changes in both auditory and visual weights were evident.

First, the early onset of these changes in audio‐visual sensory weights support the notion of low‐level and short‐latency multisensory interactions (Giard & Peronnet, 1999; Foxe *et al*., 2000; Molholm *et al*., 2002; Murray *et al*., 2005; Sperdin *et al*., 2009; Cappe *et al*., 2010). Second, our finding that visual and auditory weights scaled with reliability at different latencies during the trial is noteworthy. While visual weights were affected early (< 100 ms), auditory weights increased with decreasing reliability of the visual stimulus later (around 150 ms). This temporal dissociation of visual and auditory weight changes with reliability could reflect the adaptive nature of multisensory integration during this paradigm. Perhaps visual encoding is adjusted at short latencies and in a bottom‐up (i.e. sensory driven manner) to cope with trial‐by‐trial changes in visual sensory reliability; in contrast, auditory encoding may be adjusted only later (possibly as result of top‐down processes) to meet the increased demands for representing the unreliable sensory environment. It is also important to keep in mind that auditory reliability was fixed throughout the experiment, while visual reliability varied unpredictably. Hence, it could make sense for the brain to adapt to the visual modality on a trial‐by‐trial basis, before adjusting processing across the auditory modality subsequently, for example via the spread of attention across sensory modalities (Talsma *et al*., 2010; Donohue *et al*., 2013).

#### Early correlates of perceptual weighting

We were able to dissociate neural correlates related to perceptual weighting from correlates related to sensory reliability as not every subject attributed perceptual weights in a statistically optimal manner. Hence, the scaling of sensory representations in proportion to the physical reliability of the sensory input and the correlation of neural with perceptual weights are computationally distinct, and so reflect different aspects of the sensory‐perceptual cascade.

We found that neural correlates of the perceptual weighting process emerged early in the trial, at 120 and 204 ms after stimulus onset. These effects in neural signals – while somewhat later than the onset of modulations in the behaviour – are still earlier than expected, and a long time before the perceptual choice at the end of the trial. It remains unclear whether these perceptual weights are adjusted on each trial individually, and in response to the experienced sensory reliabilities, or whether they are at least in part already established based on task‐context in a predictive manner even before stimulus onset. Future work is required to elucidate the precise neural correlates of these perceptual weights and how different brain regions contribute to establishing the perceptual integration process.

#### Localising these effects in space and time

The temporal organisation and localisation of the reliability and perceptual weighting effects in three clusters showed distinct patterns of topographies and sources. At the earliest window (84–132 ms), effects of sensory reliability and perceptual weights were associated with occipital scalp topographies and source activity emerging from early visual and temporal areas. At the slightly later time point (156–228 ms), effects were associated with activity over central electrodes (consistent with activations including prominent contributions from auditory cortex) and source activity from temporal and parietal regions. Finally, at the latest window (252–288 ms), effects were associated with activity over posterior and central regions, and again source activity possibly originating from occipital and temporal regions. While the source localisation of the relevant EEG components was quite distributed, our results fit with the notion that earliest effects arise from occipital sensory regions and are followed by activity in the temporal and parietal lobe.

This evolving pattern of activation lends support to the idea that early sensory and parietal regions encode sensory cues and represent the integrated evidence weighted by the relative reliability and scaled by task‐demands and relevance. This complements existing findings from fMRI work showing multisensory interactions occurring along primary sensory and parietal areas in response to changing reliability. For example, Helbig *et al*. (2012) found that BOLD responses in both primary somatosensory and the superior parietal lobe increased when visual reliability decreased during a visual‐tactile task. Beauchamp *et al*. (2010) also studied visual‐tactile integration and reported that the strength of functional connections increased between somatosensory and intraparietal sulcus (IPS) for reliable somatosensory stimuli, but increased between visual and IPS for more reliable visual stimuli. Finally, Rohe & Noppeney (2015, 2016), showed that primary sensory areas encoded the spatial location of cues during an audio‐visual task while early parietal areas (IPS1‐2) represented the reliability weighted signals. Taken together with this prior literature, our results support the idea that sensory reweighting is an evolving and hierarchical process, with multisensory interactions emerging along the sensory pathway in primary sensory and parietal areas. Yet our results add a temporal dimension to these processes and demonstrate that effects related to external sensory reliability and perceptual weighting emerge at slightly different times and from distinct brain regions.

**Abbreviations**
AAL: Automatic Anatomical Labelling
AUD: Auditory
AVH: Auditory high, visual high condition
AVL: Auditory low, visual low condition
BOLD: Blood Oxygen Level Dependent
CRT: Cathode Ray Tube
EEG: Electroencephalography
fMRI: functional Magnetic Resonance Imaging
MNI: Montreal Neurological Institute
MRI: Magnetic Resonance Imaging
ROC: Receiver Operator Characteristic
SNR: Signal‐to‐Noise Ratio
VH: Visual High
VL: Visual Low

## Conflict of interest

We have no conflict of interest or competing interests to disclose.

## Author contributions

S.C.B. and C.K. carried out conception, design and implementation of experiment; S.C.B. collected data; S.C.B., S.J.K. and C.K. analysed data; S.C.B and C.K. interpreted results of experiments; S.C.B, S.J.K, C.K. wrote the manuscript.

## Data accessibility

Data/code is accessible on the Open Science Framework: https://osf.io/dyh9m/. An earlier version of this paper has been uploaded to BioRxiv (https://doi.org/10.1101/116392).

## Supporting information

Fig. S1. Performance and perceptual weighting across days.

Fig. S2. Neural weights and scalp topographies underlying the six time epochs that showed a significant effect of reliability.

Table S1. Calibration block thresholds.
