# Supplementary material for: Neural correlates of multisensory reliability and perceptual weights emerge at early latencies during audio‐visual integration
Source: Eur J Neurosci. 2017 Oct 25;46(10):2565–77. doi: 10.1111/ejn.13724 (PMC5725738; doi:10.1111/ejn.13724)
Supplement: Supplementary file 3 — Table S1. Calibration block thresholds. [file EJN-46-2565-s004.docx]

**Supplementary Figure 1. Performance and Perceptual Weighting across Days.** A) Single-subject performance (overall performance score) on each experimental session. B) Single-subject auditory weight difference (auditory low – auditory high) on each experimental session.





Supplementary Figure 2. Neural weights and scalp topographies underlying the six time epochs that showed a significant effect of reliability. Topographies marked with * represent R >0.6 and p<0.005 for correlations between scalp topographies. For each epoch the difference in neural weights (AVH_AUD_-AVH_VIS_)-(AVL_AUD_-AVL_VIS_) was calculated and correlated, * represent R>0.6 and p<0.001.

Supplementary Table 1 | Calibration Block Thresholds.

|  | Day 1 | | |  | Day 2 | | |  | Day 3 | | |  | Day 4 | | |
| --- | --- | --- | --- | --- | --- | --- | --- | --- | --- | --- | --- | --- | --- | --- | --- |
|  | AUD | VH | VL |  | AUD | VH | VL |  | AUD | VH | VL |  | AUD | VH | VL |
| S01 | 83.1 | 26.2 | 14.5 |  | 80.2 | 34.3 | 12.7 |  |  |  |  |  |  |  |  |
| S02 | 92.0 | 32.6 | 10.2 |  | 97.1 | 51.1 | 27.4 |  | 98.3 | 33.4 | 10.9 |  |  |  |  |
| S03 | 78.3 | 74.2 | 20.8 |  | 96.6 | 70.1 | 22.3 |  | 98.3 | 29.5 | 12.4 |  | 90.2 | 76.0 | 23.8 |
| S04 | 93.3 | 25.6 | 12.7 |  | 75.0 | 11.8 | 9.4 |  | 91.6 | 59.1 | 19.6 |  | 96.6 | 80.0 | 29.5 |
| S05 | 86.7 | 25.3 | 10.9 |  | 80.0 | 89.0 | 22.0 |  | 95.0 | 70.0 | 19.0 |  | 90.0 | 56.2 | 13.3 |
| S06 | 88.3 | 33.1 | 13.3 |  | 93.3 | 37.0 | 20.5 |  | 85.0 | 29.2 | 16.0 |  | 95.0 | 82.0 | 11.5 |
| S07 | 98.3 | 64.3 | 18.4 |  | 94.2 | 84.0 | 19.6 |  | 100 | 40.0 | 11.2 |  |  |  |  |
| S08 | 76.7 | 15.3 | 11.1 |  | 73.3 | 25.6 | 10.0 |  |  |  |  |  |  |  |  |
| S09 | 86.6 | 33.1 | 10.6 |  | 88.3 | 70.0 | 40.0 |  |  |  |  |  |  |  |  |
| S10 | 76.6 | 71.6 | 11.8 |  | 66.6 | 12.7 | 8.4 |  |  |  |  |  |  |  |  |
| S11 | 71.6 | 13.9 | 7.0 |  | 91.6 | 87.0 | 25.0 |  |  |  |  |  |  |  |  |
| S12 | 85.0 | 48.4 | 23.2 |  | 91.6 | 65.8 | 18.7 |  |  |  |  |  |  |  |  |
| S13 | 85.0 | 78.2 | 13.3 |  | 73.3 | 22.6 | 7.0 |  |  |  |  |  |  |  |  |
| S14 | 98.3 | 25.0 | 11.5 |  | 100 | 40.6 | 11.8 |  |  |  |  |  |  |  |  |
| S15 | 73.3 | 47.2 | 13.6 |  | 88.3 | 71.0 | 16.0 |  |  |  |  |  |  |  |  |
| S16 | 85.0 | 71.0 | 29.8 |  | 81.6 | 25.0 | 10.3 |  |  |  |  |  |  |  |  |
| S17 | 90.0 | 70.0 | 19.6 |  | 88.3 | 10.3 | 4.0 |  |  |  |  |  |  |  |  |
| S18 | 98.3 | 73.7 | 16.3 |  | 97.6 | 43.9 | 16.9 |  |  |  |  |  |  |  |  |
| S19 | 83.3 | 25.6 | 14.0 |  | 90.0 | 81.0 | 17.8 |  |  |  |  |  |  |  |  |
| S20 | 84.3 | 12.6 | 6.0 |  | 85.0 | 41.2 | 19.6 |  |  |  |  |  |  |  |  |

AUD column contain overall auditory performance score (% correct). VH and VL columns contain threshold values (contrast value) for signal to noise ratio (SNR) for high and low reliability visual stimuli respectively.
