# Supplementary material for: Neural correlates of multisensory reliability and perceptual weights emerge at early latencies during audio‐visual integration
Source: Eur J Neurosci. 2017 Oct 25;46(10):2565–77. doi: 10.1111/ejn.13724 (PMC5725738; doi:10.1111/ejn.13724)
Supplement: Supplementary file 4 [file EJN-46-2565-s002.pdf]

EJN European Journal of Neuroscience FENS Federation of European Neuroscience Societies

Neural Correlates of Multisensory Reliability and Perceptual Weights Emerge at Early Latencies during Audio-visual Integration

Stephanie C. Boyle, Stephanie J. Kayser & Christoph Kayser

Review timeline:

Submission date:	17 March 2017
Editorial Decision:	09 May 2017
Revision received:	05 July 2017
Editorial Decision:	21 August 2017
Revision received:	11 September 2017
Accepted:	18 September 2017

Editor: Sophie Molholm

1st Editorial Decision 09 May 2017

Dear Dr. Kayser,

Your manuscript was reviewed by external reviewers as well as by the Section Editor, Dr. Sophie Molholm, and ourselves.

Both reviewers are enthusiastic about the work and highlight its novelty and significance. At the same time, they raise a number of questions that will need to be addressed, and additional points that require discussion. Reviewer 1 also suggests additional analyses to strengthen interpretation of the data.

We also note the following points:
- Please ensure that the reporting of statistical data adheres to EJN guidelines (Author Instructions).
- Fig 4: grey dashed lines are difficult to see probably will not print well.
- Table 3: some p values given as 0

If you are able to respond fully to the points raised, we would be pleased to receive a revision of your paper within 12 weeks.

Thank you for submitting your work to EJN.

Kind regards,

Paul Bolam & John Foxe
co-Editors in Chief, EJN

Reviews:

Reviewer: 1 (Tim Rohe, University of Tübingen, Germany)

Comments to the Author
The authors report a study on the neural correlates of reliability-weighted multisensory integration, focusing on the temporal evolution of weighting audiovisual event rates using EEG. In line with previous reports on multisensory integration already at early stages of cortical processing, the authors find evidence of early reliability-weighted integration, presumably residing in sensory cortices.
The experiment is well designed and carefully analyzed and the results appear very interesting and novel because even though reliability-weighted integration is well investigated at the psychophysical levels, its neural underpinnings and especially its temporal evolution remain unclear. However, several major and minor points should be revised:

Major points:

1. Using psychometric analysis, the study fails to show optimal (according to the MLE model) reliability-weighted integration which has already been shown in numerous previous studies: subjects neither demonstrate a multisensory benefit (i.e. smaller multisensory perceptual threshold than either unisensory modality) nor significant reliability-dependent re-weighting of the multisensory signals (at the group level) leading to a low correlation of predicted and observed weights. This is even more surprising because the authors nicely titrated individual visual reliability levels to match unisensory visual and auditory reliabilities which should maximize the multisensory benefit according to MLE equations. In other words, the subjects do not fully integrate the signals to benefit from their redundancy. On the other hand, the authors report early reliability-related effects in different analyses of behavioral and EEG data. This paradoxical result leaves several interpretations open: First, is it a methodological difference? The psychometric analysis uses relative weight indices (i.e. both weights sum to 1) while the latter analyses uses absolute auditory and visual weights from logistic regression. Further analyses which aim at making both analysis types more comparable could clarify this, e.g. the authors could compute time-resolved relative weights using shifts of PSEs (i.e. equation 2) after fitting psychometric functions to the output of the logistic regression models (fitted to behavioral and EEG decoded data). Alternatively, the authors could also derive a relative weighting index from their logistic regression weights, e.g. by computing a relative weight as atan(beta_A / beta_B). Second, why does the brain first integrate the signals depending on their reliability but later, at the perceptual decision stage, seems to decide for an auditorily dominated percept? Obviously, a ‘forced fusion’ optimal integration model does not fully describe subjects perceptions, and the paradoxical result suggests additional later processes: One possibility could be Bayesian causal inference (Koerding et al., 2007) where a reliability-weighted average is combined with a unisensory percept depending on whether the observer perceived the signals as stemming from a single or separate sources. According to this model, the experimental setup could have led subjects not to assume a common source with certainty, where full reliability-weighted integration would have been suboptimal. The authors should analyze and discuss their data in more detail to explain this interesting, but paradoxical result.

2. Testing of EEG decoded weights in Fig. 3B/C: I find the presentation of the neural weights a bit confusing because the same data is presented twice, once from the perspective of auditory vs. visual weights (3B) and once from the perspective of visual reliability. To assess reliability-weighting, one actually needs all four plots together (as shown in 2F) which then allows to see whether a reduction of visual reliability leads to an increase of the visual and a decrease of the auditory weight (the authors could also use shaded error bars to make such a figure less crowded). In addition to testing several simple effects, the authors should also use the inherent 2x2 design to test this prediction as an interaction effect of weight x reliability which they then actually use to correlate with the psychometric reliability-reweighting effect (in 3D). The top panels of Fig. 4 suggest that such a pattern is indeed present. Further, auditory dominance could be tested as a main effect of weight across the reliability levels. Finally, in their interpretations the authors seem to neglect a significant stronger visual influence at a later epoch (around 500-550ms), what could be the reason for this finding? It is a bit puzzling because the decoder performance reaches chance level > 400ms, so how can the random output of the decoder be predicted by visual signals?

3. Source localization: The authors report source localization results without reporting whether the correlation between source signals and discriminant output reached significance in the clusters. Inferential statistics and more specific information on cluster locations would strengthen these descriptive results. Further, some kind of multiple comparison correction would be necessary given ~11000 grid points.

4. Feedback: Why did the authors chose to give trial-wise feedback? I’m not aware that many studies on the MLE model use this kind of feedback, and suppose this could have induced some kind of learning effects? How was objective feedback defined in incongruent trials where it is a priori not defined whether the auditory or the visual signals should be compared to the standard? I assume the average of both signals was used as a reference, but could it then be that subjects learned to integrate the signals with about equal weights due to this type of feedback? This could also explain the lack of the reliability-weighting effect at the psychophysical level. Maybe the authors find a different reliability-weighting effect if they only use the first part of their data where learning effects should have been weaker.

Minor points:

5. Was visual reliability manipulated randomly in each trial or block-wise? This should be stated clearly in the methods section. In the latter case, any reliability-related effects could arise from different cognitive sets or expectation/top-down attention.

6. Logistic regression on behavioral and decoded event rate judgments: In which time bins was the accumulated rate defined to build the regressors?

7. Definition of the reliability influence within the logistic regression model (equation 3): I don’t fully understand the formalization of this influence (what do the brackets mean?). I assume that the authors computed the interaction effect of reliability and weight, i.e. the difference of the difference of auditory and visual weights for both reliability levels. This could be formulated less ambiguously.

8. Linear discriminant analysis on EEG data: The authors write that the analysis was done in sliding

time windows of 55ms, so I assume that the design matrix Xt contained concatenated scalp topographies from several 5ms time points? Please clarify.

9. Manipulation of the event rate: The authors write that the single events were created by random pauses of 48 or 96ms. Where these pauses used to create different event rates on average over the 900ms stimulus periods? The authors could clarify this.

Reviewer: 2 (Ana Francisco, Albert Einstein College of Medicine, USA)

Comments to the Author
Boyle and colleagues investigated the neural correlates of audio-visual cue weighting using a rate discrimination task with EEG based neuroimaging, single-trial decoding, and linear modeling. Due to the use of different and extensive analyses, the Authors report a broad set of findings. Briefly: a) neural activity was modulated by sensory reliability early on; b) neural correlates of perceptual weights emerged shortly after stimulus onset, but before a decision was made; and c) the EEG correlates of sensory reliability and perceptual weights were localized to early sensory cortical and parietal brain areas, respectively. Though some of the results presented were expected (e.g., performance was better for high vs. low reliability), the study offers some novelty in that it adds a temporal dimension to the weighting process. This is a thorough study that constitutes an important contribution to the field. I ask, nevertheless, for some clarifications.

1)Response choices included two possibilities, both indicative of inequality. However, in some of the trials, the experimental and the standard streams were equal. Do the Authors agree that this choice might have resulted in a bias towards inequality, which could have impacted perception? Could this have had an influence in the results?

2)Visual events were presented in noise, but auditory events were presented in silence. Additionally, only the visual modality was manipulated. The Authors should justify these choices.

3)Were the auditory and visual stimuli used during the auditory and visual calibration blocks the same stimuli used in the experimental blocks?

4)On page 7 (lines 1-8) the Authors describe the auditory and the visual calibration blocks. An overall performance score was calculated for the auditory stimuli and the visual data were fit with psychometric functions. It would be good to present these results.

5)Why were the data not down-sampled by an integer factor?

6)Could the overall bias towards the auditory modality be simply explained by how much more reliable the auditory information was (since no noise was used)?

7)Participants did not systematically follow the behavioral pattern predicted by Bayesian models of multisensory integration. This finding lacks discussion.

8)Implications of the findings for the models of multisensory integration should be discussed.

Authors' Response 05 July 2017

Reply: We would like to thank the reviewers and editors for their positive and constructive comments. To address these we have performed additional analyses and made significant changes to our discussion section as per the reviewers' suggestions.

Page numbers have been added to assist with locating the changes. Additions/changes to the manuscript have been underlined and coloured in blue to highlight.

Overall Comments to address:

Both reviewers are enthusiastic about the work and highlight its novelty and significance. At the same time, they raise a number of questions that will need to be addressed, and additional points that require discussion. Reviewer 1 also suggests additional analyses to strengthen interpretation of the data.

We also note the following points:

EJN European Journal of Neuroscience FENS Federation of European Neuroscience Societies

1. Please ensure that the reporting of statistical data adheres to EJN guidelines (Author Instructions).

Reply: We have worked through the Statistics checklist pdf provided in the Author Instructions. Data and code are currently being organised and will be uploaded to the open science framework webpage (Project page created here: https://osf.io/dyh9m/), or can be uploaded to Figshare if this is the preferred option for EJN. If accepted, we will ensure the upload is complete before publication.

2. Fig 4: grey dashed lines are difficult to see probably will not print well.

Reply: We have made the lines darker in each figure.

3. Table 3: some p values given as 0.

Reply: We had originally noted in the figure caption that p-values less than 10^-3 were abbreviated as zero. However, we have removed these and added p<0.001 to the tables instead.

Reviewer: 1

The authors report a study on the neural correlates of reliability-weighted multisensory integration, focusing on the temporal evolution of weighting audiovisual event rates using EEG. In line with previous reports on multisensory integration already at early stages of cortical processing, the authors find evidence of early reliability-weighted integration, presumably residing in sensory cortices. The experiment is well designed and carefully analyzed and the results appear very interesting and novel because even though reliability-weighted integration is well investigated at the psychophysical levels, its neural underpinnings and especially its temporal evolution remain unclear. However, several major and minor points should be revised:

Major points:

1. Using psychometric analysis, the study fails to show optimal (according to the MLE model) reliability-weighted integration which has already been shown in numerous previous studies: subjects neither demonstrate a multisensory benefit (i.e. smaller multisensory perceptual threshold than either unisensory modality) nor significant reliability-dependent re-weighting of the multisensory signals (at the group level) leading to a low correlation of predicted and observed weights. This is even more surprising because the authors nicely titrated individual visual reliability levels to match unisensory visual and auditory reliabilities which should maximize the multisensory benefit according to MLE equations. In other words, the subjects do not fully integrate the signals to benefit from their redundancy. On the other hand, the authors report early reliability-related effects in different analyses of behavioral and EEG data. This paradoxical result leaves several interpretations open:

Reply: To address these points, we have separated the reviewers' comments out below with separate replies noted underneath.

First, is it a methodological difference? The psychometric analysis uses relative weight indices (i.e. both weights sum to 1) while the latter analyses uses absolute auditory and visual weights from logistic regression. Further analyses which aim at making both analysis types more comparable could clarify this, e.g. the authors could compute time-resolved relative weights using shifts of PSEs (i.e. equation 2) after

fitting psychometric functions to the output of the logistic regression models (fitted to behavioral and EEG decoded data).

Reply: The neural weights (the output of the logistic regression model) are calculated using all trials of all rates (that are not equal to the comparison stream rate), with each modality reliability level modelled separately as a predictor (4 predictors: AH,AL,VH,VL). This makes it impossible to fit psychometric curves to the logistic output separately for each rate and reliability level to then compute time-resolved weights using Eqn 2, as per the reviewer's suggestion.

However in line with the reviewer's suggestion, we have tried to fit psychometric curves to the output of the decoder (the discriminant signal Y) rather than the output of the regression model. Unfortunately, this did not show robust results, and for some time points the curve provided a very poor fit, presumably due to the noise arising from relying on fewer trials than the regression approach (i.e. separating trials by rate/reliability) at each time point. Therefore, we feel that the regression model generates the best estimate of neural weights at each time point.

Alternatively, the authors could also derive a relative weighting index from their logistic regression weights, e.g. by computing a relative weight as atan(beta_A / beta_B).

Reply: We calculated this measure as shown in the figures below. However it limits us from making comparisons between high and low reliability (when beta low/beta high) or between modalities (when beta auditory/beta visual) directly, as with this approach one can't derive separate weights for each level of reliability or modality.

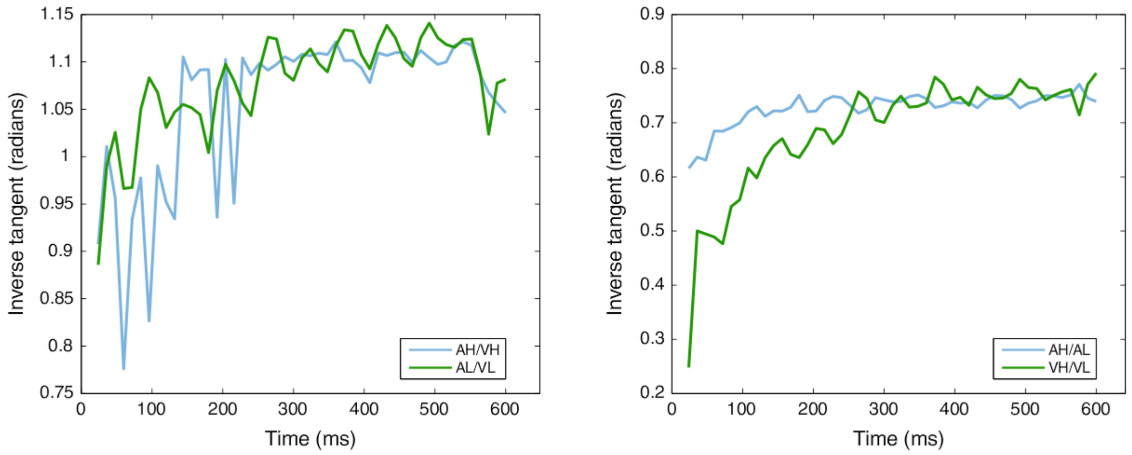

In conclusion, we feel that because the precise neural origin of the EEG components related to sensory reliability (and hence the respective generators of auditory and visual contributions to these), remain unclear, we cannot assume that the auditory and visual neural weights (for each reliability) normalize to a fixed sum of one.

And as our goal was to relate the behavioural weights derived from the regression to the neural weights, we feel it is more important to keep these two measures comparable, rather than attempting to make the two behavioural measures comparable. Uncovering why subjects

psychometric performance was inconsistent with the optimal integration model is an interesting question, but not one we can focus on in this project. Rather, we decided to exploit the mismatch between perceptual weighting and sensory reliability we find to explore the evolution of the neural signals for each of these.

In line with the reviewer's comment we have extended the discussion as summarised below:

(1) Subject's failure to demonstrate multisensory benefit: We acknowledge that we find reliability weighting without optimal integration in the discussion, and have strengthened this section per the reviewer comments by including a figure with the individual subject threshold data plotted (rather than a group level psychometric curve) in Figure 2C. We have also added the lack of threshold differences into the discussion section (Page 23, 548-551).

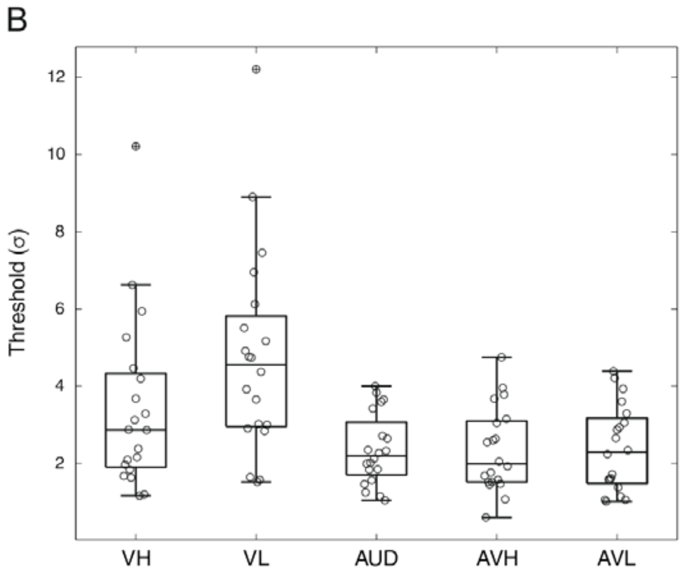

(2) Perceptual Weighting/Methodological difference: We have added the points discussing the reasons that we may not have found reliability dependent weighting at a group level using the integration model, but we did find reliability dependent weighting at early time points using a regression model raised by the reviewer into our discussion. This has been added in the section "Perceptual Weights", (Page 22, 521 - Page 24, 590) and includes:

(a) Pointing out that it could be due to methodological differences (as per reviewer 1's suggestion). (Page 22, 535- 536)

(b) that it could arise because the optimal integration model does not calculate a set of weights for each time point in a trial, and therefore may not be the best way to capture the time-dependence of relevant effects. (Page 22, 536-Page 23, 545).

(c) that the lack of reliability weighting seen in the integration model could be the result of the model not taking into account the bias towards the auditory modality; previous work (Butler et al., 2010; Battaglia et al., 2003) has shown that adding a prior to account for biases towards modality dominance provides a better fit to the data when searching for optimality effects. (Page 24, 567-576).

(d) but we also acknowledge that the significant correlation between reliability effects in the two types of behavioural weights suggest that the difference in these approaches is possibly more gradual than conceptual. (Page 23, 542-545).

2. Second, why does the brain first integrate the signals depending on their reliability but later, at the perceptual decision stage, seems to decide for an auditorily dominated percept?

Obviously, a 'forced fusion' optimal integration model does not fully describe subjects perceptions, and the paradoxical result suggests additional later processes: One possibility could be Bayesian causal inference (Koerding et al., 2007) where a reliability-weighted average is combined with a unisensory percept depending on whether the observer perceived the signals as stemming from a single or separate sources. According to this model, the experimental setup could have led subjects not to assume a common source with certainty, where full reliability-weighted integration would have been suboptimal. The authors should analyze and discuss their data in more detail to explain this interesting, but paradoxical result.

Reply: Behaviourally we find auditory overweighting throughout the trial. Therefore, we feel that the auditory dominated percept does not arise at a later perceptual decision stage, but is evident at early points during the trial (see Behavioural regression weights). This is also apparent in the EEG data (Figure 4B), although auditory dominance is more pronounced in the low reliability condition. We had touched on this point of auditory dominance in our discussion, but have expanded this section further to include the relevant points the reviewer states above (Page 23, 548-551).

We also acknowledge that this paradoxical result is interesting in terms of causal inference in relation to behaviour, and have expanded the discussion to include a more explicit link of the causal inference model (Page 24, 577-590). However, this question and analysis (e.g. fitting causal inference models and studying the underlying brain dynamics) is an entirely new project rather than an addition to this existing one, and is better suited for future work. In particular, it would be technically impossible to reliably fit a full causal inference model to the present data given that only a limited range of audio-visual discrepancies were presented during the experiment (Page 24, 585-590).

3. Testing of EEG decoded weights in Fig. 3B/C: I find the presentation of the neural weights a bit confusing because the same data is presented twice, once from the perspective of auditory vs. visual weights (3B) and once from the perspective of visual reliability. To assess reliability-weighting, one actually needs all four plots together (as shown in 2F) which then allows to see whether a reduction of visual reliability leads to an increase of the visual and a decrease of the auditory weight (the authors could also use shaded error bars to make such a figure less crowded).

Reply: We re-created the figure using shaded error bars and all four graphs on one figure. However, this makes it difficult to distinguish the auditory from visual signals (even with shaded error bars), as well as the significant time points where there was a difference for each reliability level (high vs. low reliability for auditory and visual separately) from the significant time points where there was a difference between modality weighting (auditory vs. visual weighting). We found it difficult to interpret and see differences even when we moved the significant points to below the plotted lines:

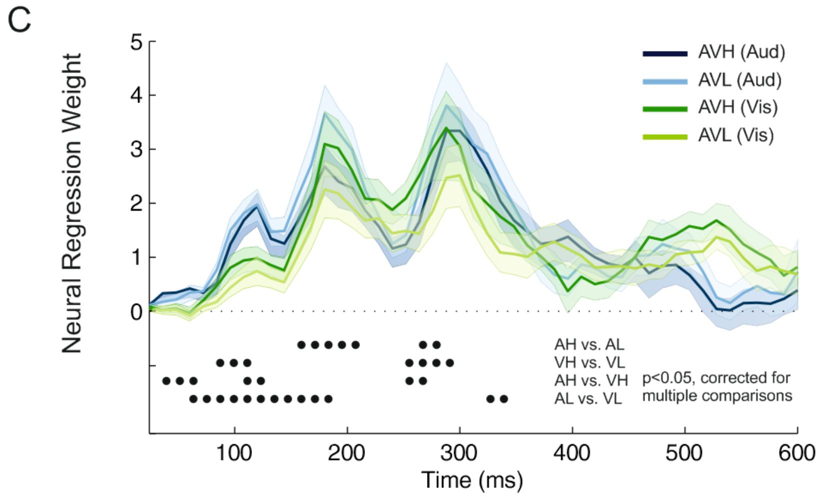

We hence decided to stick to the redundant, but visually more transparent presentation in four separate axes. To make it clearer that the four graphs reflect different effects (although they present the same data twice), we changed the figure titles to more accurately indicate the comparisons, and included a sentence in the figure legend making it more clear what is being displayed. Additionally, we removed the error bars and included shaded error bars on all graphs to make the graphs less noisy, as per the reviewer's suggestion.

Old plot:

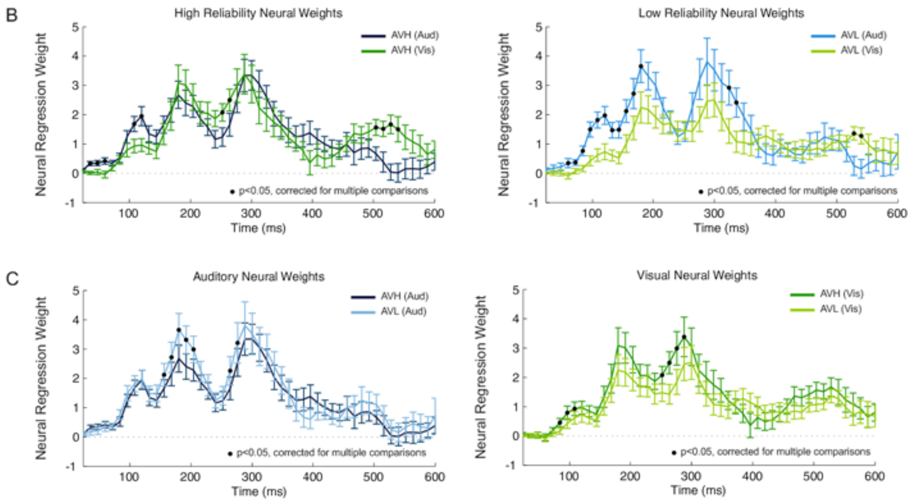

EJN European Journal of Neuroscience FENS Federation of European Neuroscience Societies

New plot:

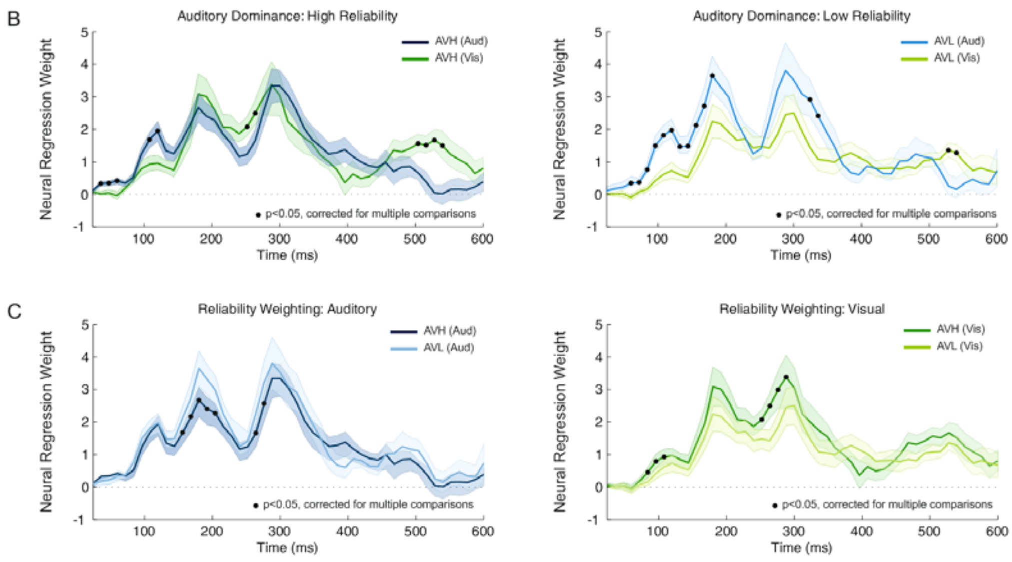

5. In addition to testing several simple effects, the authors should also use the inherent 2x2 design to test this prediction as an interaction effect of weight x reliability which they then actually use to correlate with the psychometric reliability-reweighting effect (in 3D). The top panels of Fig. 4 suggest that such a pattern is indeed present. Further, auditory dominance could be tested as a main effect of weight across the reliability levels.

> Reply: We had originally considered testing the full 2x2 design. However, given that auditory and visual weights are independent and of possibly different scales as they were not assumed to normalize to one, and given that experimentally reliability was manipulated only for one sensory modality, we did not anticipate to find a main effect or reliability across both modalities. Rather, we decided to directly test our specific hypotheses of each individual weight to change with reliability. As a result, we tested effects of reliability and modality dominance separately.

6. Finally, in their interpretations the authors seem to neglect a significant stronger visual influence at a later epoch (around 500-550ms), what could be the reason for this finding? It is a bit puzzling because the decoder performance reaches chance level > 400ms, so how can the random output of the decoder be predicted by visual signals?

> Reply: We had originally not made interpretations about the visual effect later in the trial for the reasons the reviewers noted, in particular as the decoder reached chance after 400ms. We agree that it could lead to interpretations that could be confusing and therefore we have for all analyses restricted statistical testing and figures to the time period from stimulus onset to 400ms.

7. Source localization: The authors report source localization results without reporting whether the correlation between source signals and discriminant output reached significance in the clusters. Inferential statistics and more specific information on cluster locations would strengthen these descriptive results. Further, some kind of multiple comparison correction would be necessary given ~11000 grid points.

Reply: We have added results that show the inferential statistics, corrected for multiple comparisons in Table 4, and have added a line noting this in the results (Page 21, 497).

8.

Reply: To address this comment, we have separated the points below.

Feedback: Why did the authors chose to give trial-wise feedback?

Reply: We chose to give trial-wise feedback to keep subjects engaged with the task over the course of a long EEG session. The session was long (~3 hours per session, with subjects completing multiple sessions across days) to accommodate for appropriate inter-trial intervals and baseline periods necessary for EEG recording.

We have added this explanation of why we chose to give feedback to the paper (Page 9, 196-201)

I'm not aware that many studies on the MLE model use this kind of feedback…

Reply: We deemed it acceptable to give participant feedback based on previous work that has used feedback when testing for reliability cue weighting and which found no difference between participants who received feedback and those who did not (Raposo et al., 2010; Sheppard et al., 2012). We have added this to the manuscript (Page 9, 196-199).

and suppose this could have induced some kind of learning effects?

Reply: To test for confounding learning effects (even though we had tried to control for such a confound by carrying out individual subject calibration blocks at the start of each new day), we had originally analysed the behavioural data separately by day. This showed that subjects did not necessarily improve their performance across days (see plot below). We have added this plot to the Supplementary Materials (Fig.1A).

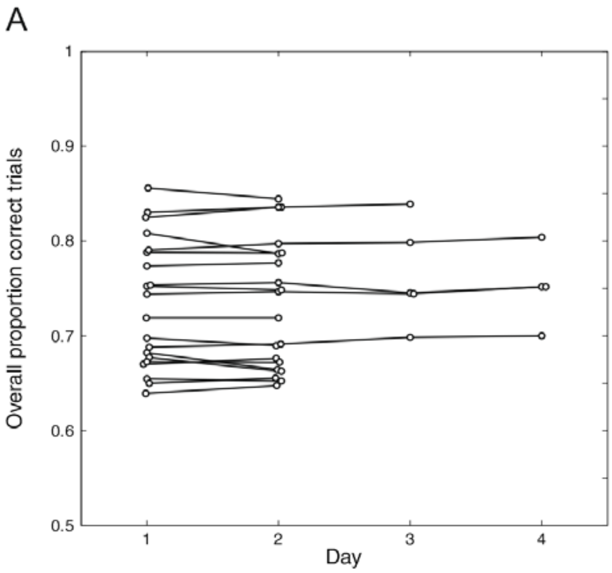

9.

Reply: To address this comment, we have separated the points below.

How was objective feedback defined in incongruent trials where it is a priori not defined whether the auditory or the visual signals should be compared to the standard?

Reply: In this experiment subjects were not aware that the stimulation rate of the auditory and visual streams was different in audio-visual trials. They were instructed to compare which stream had a higher number of "events", where an "event" was defined as an auditory click, visual flash, or audio-visual click and flash presented together. Therefore, there were no cases where it was not defined whether the auditory or visual signals should be compared to the standard.

I assume the average of both signals was used as a reference, but could it then be that subjects learned to integrate the signals with about equal weights due to this type of feedback?

Reply: To generate feedback for the incongruent conditions the average rate of the auditory and visual streams was used. However, we were not providing specific feedback about each individual rate, only about whether the subject's response was correct or incorrect based on the comparison to a standard event rate of 11Hz. For example, if the visual rate was 8Hz, and the auditory rate was 10Hz, the average rate was 9Hz. In this case, subjects would be provided feedback that they were correct if they responded that the second stream had a higher event rate; in such a trial, this feedback is correct for both the visual and the auditory rate. The same applies to higher event rates (e.g. where the visual is 12Hz and the auditory is 14Hz). Therefore, subjects' decisions about these rates should not be influenced towards equal weighting based on the feedback.
In cases where the average event rate is equal to the comparison stream rate (e.g. visual rate is

10Hz and auditory rate is 12Hz and the average rate is 11Hz) feedback was randomly generated, with the aim of reducing bias.

In the case where the event rate crosses the comparison stream boundary (e.g. where visual rate is 9Hz and auditory is 11Hz, with the average rate being 10Hz), we agree that this could potentially mislead subjects, but this is a small number of trials (8%), and this was symmetric to auditory>visual (4%) and visual>auditory (4%) trials.

Finally, as noted above, past work has shown feedback does not affect subject weighting on a reliability task (Raposo, Sheppard, Schrater, & Churchland, 2012; Sheppard, Raposo, & Churchland, 2013). This is further discussed below in relation to the next comment:

This could also explain the lack of the reliability-weighting effect at the psychophysical level. Maybe the authors find a different reliability-weighting effect if they only use the first part of their data where learning effects should have been weaker.

Reply: We did not find any performance difference across days (see: response to comment 8 above), and so we did not originally calculate the perceptual weights for each day separately. In response to the reviewer's comment, we did however calculate a set of perceptual weights for each day, and this showed that weighting strategies were not necessarily different in later days than earlier days as evidenced by the plot below. We have also included in the Supplementary Materials as Figure 1B):

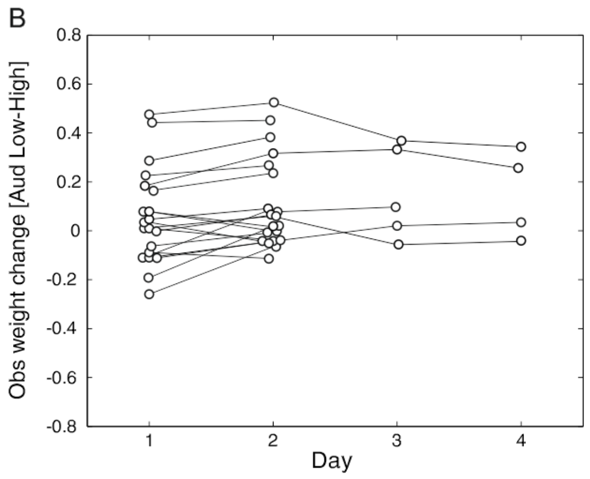

10. Was visual reliability manipulated randomly in each trial or block-wise? This should be stated clearly in the methods section. In the latter case, any reliability-related effects could arise from different cognitive sets or expectation/top-down attention.

Reply: All reliability levels (auditory/visual) were set during the threshold blocks at the beginning of the session, and held constant throughout that block. In each block all conditions (visual high, visual low, auditory high, audio-visual high [all conflict levels], audio-visual low [all conflict levels]) were presented in a random order. This created trial-by-trial variability in the modality, rate, and reliability of the stimulus on each trial within a block. We have included instructions to clarify this (Page 8, 181-182).

11. Logistic regression on behavioral and decoded event rate judgments: In which time bins was the accumulated rate defined to build the regressors?

Reply: Accumulated rate was calculated in 12ms time bins (our stimuli were each presented for 12ms), resulting in 75 time points (making up our stimulus stream length of 900ms). This detail has been added to the manuscript (Page 11, 261-263).

12. Definition of the reliability influence within the logistic regression model (equation 3): I don't fully understand the formalization of this influence (what do the brackets mean?). I assume that the authors computed the interaction effect of reliability and weight, i.e. the difference of the difference of auditory and visual weights for both reliability levels. This could be formulated less ambiguously.

Reply: We have updated our formulation, and tried to explain it more clearly in the text (Page 12, 274-279).

New Formulation: $D(t) = [AVH_{WAUD} - AVH_{WVIS}] - [AVL_{WAUD} - AVL_{WVIS}]$

13. Linear discriminant analysis on EEG data: The authors write that the analysis was done in sliding time windows of 55ms, so I assume that the design matrix Xt contained concatenated scalp topographies from several 5ms time points? Please clarify.

Reply: The linear discriminant analysis uses the EEG data (for each electrodes, trials) averaged over a certain time window (in this case, 55ms time window) as input. We have added a line to clarify this. (Page 13, 303-306).

14. Manipulation of the event rate: The authors write that the single events were created by random pauses of 48 or 96ms. Where these pauses used to create different event rates on average over the 900ms stimulus periods? The authors could clarify this.

Reply: The event rates were pre-determined, and the pauses randomly interspersed between the stimuli to create the flicker rates over the 900ms periods. We have added in more detail to the Methods to clarify this (Page 5, 113- Page 6, 121).

Reviewer: 2

Comments to the Author

Boyle and colleagues investigated the neural correlates of audio-visual cue weighting using a rate discrimination task with EEG based neuroimaging, single-trial decoding, and linear modeling. Due to the use of different and extensive analyses, the Authors report a broad set of findings. Briefly: a) neural activity was modulated by sensory reliability early on; b) neural correlates of perceptual weights emerged shortly after stimulus onset, but before a decision was made; and c) the EEG correlates of sensory reliability and perceptual weights were localized to early sensory cortical and parietal brain areas, respectively. Though some of the results presented were expected (e.g., performance was better for high vs. low reliability), the

study offers some novelty in that it adds a temporal dimension to the weighting process. This is a thorough study that constitutes an important contribution to the field. I ask, nevertheless, for some clarifications.

1. Response choices included two possibilities, both indicative of inequality. However, in some of the trials, the experimental and the standard streams were equal. Do the Authors agree that this choice might have resulted in a bias towards inequality, which could have impacted perception? Could this have had an influence in the results?

> Reply: We agree that forcing subjects to select whether an experimental stream of 11 Hz was higher or lower than a comparison stream of 11 Hz biases subjects towards assuming inequality. However, these trials made up only a small percentage of trials (2% of trials for each condition), and occurred randomly during the experimental block, and we tried to control for any effects of this by generating the feedback randomly so that subjects could not "learn" about the inequality. Based on these reasons, we do not feel these trials where the rate is equal to the comparison stream rate would systematically influence behaviour.
>
> Additionally, while these 11Hz trials were included for the integration model analysis to fit psychometric curves to the data, they were excluded from both regression analyses to avoid high correlation between the visual and auditory signals. They were also excluded from the decoding analysis, as the decoder needs to classify between high and low rate conditions (and cannot classify a condition where the rates were equal) and therefore equal rate trials cannot impact the results.

2. Visual events were presented in noise, but auditory events were presented in silence. Additionally, only the visual modality was manipulated. The Authors should justify these choices.

> Reply: In order to facilitate the implementation in an EEG study, which requires longer inter-trial intervals than purely behavioural studies, we had to reduce the number of experimental conditions (e.g. compared to purely behavioural studies). We therefore manipulated reliability only in one modality, similar to other neuroimaging studies (Helbig et al., 2012; Rohe & Noppeney, 2015, 2016) and neurophysiological studies (Fetsch, Pouget, DeAngelis, & Angelaki, 2012). We have added this justification to the methods section (Page 6, 139- Page 7, 144).

3. Were the auditory and visual stimuli used during the auditory and visual calibration blocks the same stimuli used in the experimental blocks?

> Reply: Yes, however in the calibration blocks we chose to use the easiest rates (8 Hz, 14 Hz) to compare to the comparison stream (11Hz). We have added this detail to the methods (Page 7, 163-166).

4. On page 7 (lines 1-8) the Authors describe the auditory and the visual calibration blocks. An overall performance score was calculated for the auditory stimuli and the visual data were fit with psychometric functions. It would be good to present these results.

> Reply: For each session, we calculated a threshold for each participant. This means that each

subject has between 2-4 psychometric curves and 2-4 auditory thresholds (depending on how many days subjects participated). Plotting this in an easily interpretable way is somewhat difficult, and so we have included a table of calibration block threshold values in the supplementary materials (Supplementary Table 1.)

5. Why were the data not down-sampled by an integer factor?

Reply: The data was down sampled using FieldTrip Software which allows to manually choose an arbitrary target sampling rate.

6. Could the overall bias towards the auditory modality be simply explained by how much more reliable the auditory information was (since no noise was used)?

Reply: Yes we do feel it can be also explained by the auditory modality being more reliable due to no noise (alongside the auditory modality being better suited for the rate discrimination task at hand). We have extended the Discussion to more explicitly acknowledge this (Page 23, 553-557).

7. Participants did not systematically follow the behavioural pattern predicted by Bayesian models of multisensory integration. This finding lacks discussion. Implications of the findings for the models of multisensory integration should be discussed.

Reply: We have expanded upon the discussion to include more discussion on literature from the field that has also found non-optimality in behavioural performance, as well as a section on how MLE/Bayesian optimal integration models can be adapted in such cases (Page 24, 577-590).

Additional changes not mentioned above:

1. Line numbers have been added to assist reviewers with locating changes.

Methods:

2. We have added a section explaining how we aimed to reduce behavioural bias on trials where the experimental stream event rate was equal to the comparison stream event rate (Page 6, 124-127).

3. We have added to the description of how many days/blocks/trials subjects completed to provide more information for readers (Page 8, 182-186).

4. We had wrongly cited Fetsch et al., 2012 as the source of Eqn (1) (predicted weights):

$$W_{AUD} = [\sigma_{VIS}^2 / [\sigma_{AUD}^2 + \sigma_{VIS}^2] \qquad (1)$$

This equation originally came from Sheppard et al., 2013.

EJN European Journal of Neuroscience FENS Federation of European Neuroscience Societies

To keep the equations in the paper consistent from Fetsch et al., 2012 we have updated Eqn 1 to:

$$W_{AUD} = 1/\sigma^2_{AUD} / [1/\sigma^2_{AUD} + 1/\sigma^2_{VIS}] \qquad (1)$$

which gives the same predicted weight output as the original equation from the Sheppard paper. We feel this adds more consistency to the methods section (Page 10, 237).

5. We have added a description of why we chose not to normalise the neural weights (Page 14, 323-329)

6. We had not included the minimum cluster size used in our comparisons. This has been added (Page 15, 364).

7. We have added a description of how effect size for cluster permutation testing was calculated (Page 15, 365-367).

Figures:

8. Figure 2C. We have changed this figure panel from the "Observed vs. Predicted Weights" scatterplot to the thresholds figure discussed in the reviewer comments. We feel this change is acceptable for three reasons: (1) the individual variation in thresholds are now extensively noted in the discussion section (in line with reviewer comments), (2) the observed vs. predicted weight comparison is not the main focus of the paper, and (3) the observed vs. predicted weight correlation results and comparisons are already included in a table.

9. Figure 2D. Ylabel changed to "Psychometric Auditory Weights (AVL-AVH)" *(from "AVL Aud – AVH Aud").*

10. Figure 2 [F/G]. Points denoting significance made thicker/easier to see.

11. Figure 2F. Error bars replaced with shaded error bars.

12. Figure 2G. Changed ylabel to "Correlations of behavioural weights (R)" (*from "R").*

13. Figure 3 [B/C/D/E]. Error bars replaced with shaded error bars.

14. Figure 3A. Changed ylabel to "Mean Discriminant Performance (Az)" *(from "Mean Classifier (Az) Performance")*.

15. Figure 3D. Ylabel changed to "Neuro-behavioural correlation (R)" *(from R value)* and made the points denoting significance easier to see.

16. Figure 4 [A/B/C top]. Removed fill from single subject plot lines (to make clearer).

17. Figure 4 [A/B/C middle and bottom]. Changed all colorbars to be same scale. Removed redundant colorbars to make space. Added labels to the colorbars.

Deletions:

1. Table I. We deleted two significant cluster time points that were incorrectly included as significant for Auditory (cluster 3: 120ms to 168ms, and cluster 4: 192ms to 276ms). This should have been only two clusters. These are now correctly reported.

EJN European Journal of Neuroscience FENS Federation of European Neuroscience Societies

2. Table III. We deleted statistical values corresponding to time points after 400ms.

2nd Editorial Decision 21 August 2017

Dear Dr. Kayser,

Your revised manuscript was re-evaluated by external reviewers as well as by the Section Editor, Dr. Sophie Molholm and ourselves. We are pleased to inform you that your manuscript is now suitable for publication in EJN. We have indicated 'minor revision' so that you can revise in accordance with reviewer 1's comments if you see fit (adding analysis suggested under point 5, and referring to Figure 2C in the results or discussion).

Please also check the reference list which contains some errors and provide the precise values of P, in accordance with EJN guidelines.

If you are able to respond fully to the points raised, we shall be pleased to receive a revision of your paper within 30 days.

Thank you for submitting your work to EJN.

Kind regards,

Paul Bolam & John Foxe
co-Editors in Chief, EJN

Reviews:

Reviewer: 1 (Tim Rohe, University of Tübingen, Germany)

Comments to the Author
The authors responded thoroughly to all points and made important new analyses and additions to the manuscript. My point-by-point responses (only point 6 might need another analysis):

Point 1:
Approach atan(beta_A / beta_V): I agree with the reviewers that one cannot sensibly compare this weight index between modalities (because atan(beta_A_VL / beta_A_VH) makes not much sense), but the effect of reliability can be compared as in their left panel where one can see a stronger visual relative weight for high visual reliability between 100-200ms and a subsequent convergence to auditory dominance. Though quite revealing, one can also infer this indirectly from the authors current figure 3B (with some visual scanning though). Moreover, the atan figure looks a bit noisy and testing the reliability effect would also require circular statistics. Thus, I agree with the reviewers to leave the current presentation of results for the sake of consistency/conciseness of the paper.
Figure 2C. A valuable addition to the results (maybe the figure should then also be referenced in the discussion or results?)

Point 2: I fully agree to the authors responses.
Point 3: I personally prefer the four-graphs figure, but admit that it is quite difficult to discern single plots, so it’s up to the author’s choice.

Point 5: Obviously, the auditory and visual weights are independent and do not sum to 1, but this does not preclude a factorial analyses of the main and interaction effects of modality and reliability. Of course, the authors can test the simple effects (within factorial cells), however, reporting the main and interaction effects (if any) would be common statistical practice and informative for the reader.

Point 6: Ok, the authors restrict their analyses to a time interval of significant decoder performance which will leave the reader less puzzled. Still interesting how a decoded producing random output in training conditions is then able to produce a signal in a different condition…or might there be some

EJN European Journal of Neuroscience FENS Federation of European Neuroscience Societies

bias in the decoder? The authors could quickly check this by a randomization approach, i.e. randomizing labels before training the decoder and then check whether the late visual influence persists (i.e. all neural regression weights should converge to 0 under randomization).

Point 7. An important addition to the original results.

P8: Ok, clearly no feedback-associated learning visible in the plot.
P9: Given these additional results, it seems unlikely that observers learnt cue weighting due to the feedback.
P10-14: Ok, the authors clarified all points.

Reviewer: 2 (Ana Francisco, Albert Einstein College of Medicine, USA)

Comments to the Author
All previous concerns were addressed excellently. I am happy to recommend acceptance of this manuscript.

Authors' Response 11 September 2017

Overall comments: Please also check the reference list which contains some errors and provide the precise values of P, in accordance with EJN guidelines.

Reply: We have fixed the errors in the reference list.

In text we refer to comparisons as being lower than a threshold value, and include references for the corresponding tables, which contain all exact statistical values. This was to make the text easier to read as there were many comparisons. We have added a line to clarify that the specific values are included in tables: Pg 16, 382-284

Reviewer: 1

Reply: We thank the reviewers for reviewing the manuscript revisions. We have made our amendments and responded to the reviewers' comments below.

Comments to the Author
The authors responded thoroughly to all points and made important new analyses and additions to the manuscript. My point-by-point responses (only point 6 might need another analyses):

Point 1:
Approach atan(beta_A / beta_V): I agree with the reviewers that one cannot sensibly compare this weight index between modalities (because atan(beta_A_VL / beta_A_VH) makes not much sense), but the effect of reliability can be compared as in their left panel where one can see a stronger visual relative weight for high visual reliability between 100-200ms and a subsequent convergence to auditory dominance. Though quite revealing, one can also infer this indirectly from the authors current figure 3B (with some visual scanning though). Moreover, the atan figure looks a bit noisy and testing the reliability effect would also require circular statistics. Thus, I agree with the reviewers to leave the current presentation of results for the sake of consistency/conciseness of the paper.

Reply: Ok.

Figure 2C. A valuable addition to the results (maybe the figure should then also be referenced in the discussion or results?)

Reply: The figure is referenced already in the results section (Page 167, Line 395).

Point 2: I fully agree to the authors responses.

Reply: Ok.

Point 3: I personally prefer the four-graphs figure, but admit that it is quite difficult to discern single plots, so it's up to the author's choice.

Reply: We thank the reviewer for the comments. We have decided to include the new four-graph figure (with the shaded error bars as per the reviewer's original suggestion). This is so the significant time points are easily discernible.

Point 5: Obviously, the auditory and visual weights are independent and do not sum to 1, but this does not preclude a factorial analyses of the main and interaction effects of modality and reliability. Of course, the authors can test the simple effects (within factorial cells), however, reporting the main and interaction effects (if any) would be common statistical practice and informative for the reader.

Reply: We appreciate that an ANOVA would provide complementary information to the results contained already in the manuscript. However, as mentioned previously, we did not originally hypothesise the existence of a main effect of modality or an interaction between modality and reliability. Consequently, we tested for the hypothesised effect of reliability at each time point across both modalities. We feel that the manuscript already contains many comparisons between conditions, and including both a new ANOVA analysis (which changes the initial hypothesis of the effects) alongside the results we already have would be. We thank the reviewer for the interesting suggestion, but on this occasion feel that adding the results of an ANOVA test alongside the originally planned tests takes away from the results we already present.

Point 6: Ok, the authors restrict their analyses to a time interval of significant decoder performance which will leave the reader less puzzled. Still interesting how a decoded producing random output in training conditions is then able to produce a signal in a different condition…

Reply: For the decoding analysis we trained the classifier to discriminate one condition (in our case, sensory rate), and tested it on different trials of the same condition. We do not ask the decoder to train on one set of conditions and produce a signal in a different condition. The decoder will produce a signal regardless of significance: LDA aims to linearly separate the EEG data at each time point and generate a one-dimensional projection in the data corresponding to the separation. This resulting projection may be good or bad but will be outputted regardless of significance. Thus, there is no reason for the decoder not to produce a signal.

Point 6, continued: or might there be some bias in the decoder? The authors could quickly check this by a randomization approach, i.e. randomizing labels before training the decoder and then check whether the late visual influence persists (i.e. all neural regression weights should converge to 0 under randomization).

Reply: We performed 1000 randomisations and here plot the resulting averaged signal:

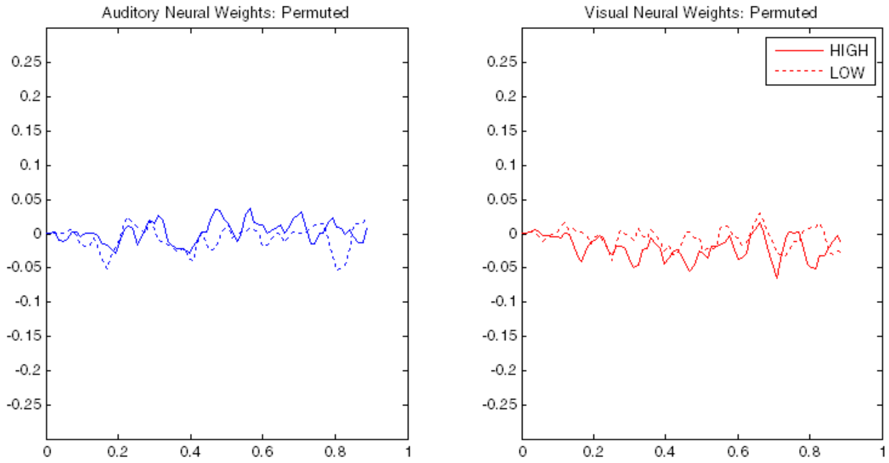

EJN European Journal of Neuroscience FENS Federation of European Neuroscience Societies

This shows that neural weights resulting from shuffling decoding labels do converge around zero. Thus we feel that the decoder is not biased and by focusing the analysis only on the time window of significant classification we have a stronger result, and thank the reviewer for the original comment.

Point 7. An important addition to the original results.

Reply: We agree, and thank the reviewer for the suggestion.

P8: Ok, clearly no feedback-associated learning visible in the plot.

P9: Given these additional results, it seems unlikely that observers learnt cue weighting due to the feedback.

P10-14: Ok, the authors clarified all points.

Reply 8-14: Ok.

Reviewer: 2

Comments to the Author
All previous concerns were addressed excellently. I am happy to recommend acceptance of this manuscript.

Reply: We thank the reviewer for reviewing the manuscript.
